# Whole-Blood 3-Gene Signature as a Decision Aid for Rifapentine-based Tuberculosis Preventive Therapy^[Author-notes fn-0003]^

**DOI:** 10.1093/cid/ciac003

**Published:** 2022-01-05

**Authors:** Hung Ling Huang, Jung Yu Lee, Yu Shu Lo, I Hsin Liu, Sing Han Huang, Yu Wei Huang, Meng Rui Lee, Chih Hsin Lee, Meng Hsuan Cheng, Po Liang Lu, Jann Yuan Wang, Jinn Moon Yang, Inn Wen Chong

**Affiliations:** Division of Pulmonary and Critical Care Medicine, Kaohsiung Medical University Hospital, Kaohsiung, Taiwan; Department of Internal Medicine, Kaohsiung Medical University Hospital, Kaohsiung, Taiwan; Department of Internal Medicine, Kaohsiung Municipal Ta-Tung Hospital, Kaohsiung, Taiwan; Graduate Institute of Medicine, College of Medicine, Kaohsiung Medical University, Kaohsiung, Taiwan; Institute of Bioinformatics and Systems Biology, National Yang Ming Chiao Tung University, Hsinchu, Taiwan; Institute of Bioinformatics and Systems Biology, National Yang Ming Chiao Tung University, Hsinchu, Taiwan; Institute of Bioinformatics and Systems Biology, National Yang Ming Chiao Tung University, Hsinchu, Taiwan; Institute of Bioinformatics and Systems Biology, National Yang Ming Chiao Tung University, Hsinchu, Taiwan; Institute of Bioinformatics and Systems Biology, National Yang Ming Chiao Tung University, Hsinchu, Taiwan; Department of Internal Medicine, National Taiwan University Hospital, Hsinchu Branch, Hsinchu, Taiwan; Department of Internal Medicine, National Taiwan University Hospital, National Taiwan University College of Medicine, Taipei, Taiwan; Division of Pulmonary Medicine and Pulmonary Research Center, Wanfang Hospital, Taipei Medical University, Taipei, Taiwan; Division of Pulmonary and Critical Care Medicine, Kaohsiung Medical University Hospital, Kaohsiung, Taiwan; Department of Internal Medicine, Kaohsiung Medical University Hospital, Kaohsiung, Taiwan; Graduate Institute of Medicine, College of Medicine, Kaohsiung Medical University, Kaohsiung, Taiwan; Department of Respiratory Therapy, Kaohsiung Medical University Hospital, Kaohsiung, Taiwan; Department of Internal Medicine, Kaohsiung Medical University Hospital, Kaohsiung, Taiwan; Graduate Institute of Medicine, College of Medicine, Kaohsiung Medical University, Kaohsiung, Taiwan; Center for Liquid Biopsy and Cohort, Kaohsiung Medical University, Kaohsiung, Taiwan; Department of Internal Medicine, National Taiwan University Hospital, National Taiwan University College of Medicine, Taipei, Taiwan; Institute of Bioinformatics and Systems Biology, National Yang Ming Chiao Tung University, Hsinchu, Taiwan; Center for Intelligent Drug Systems and Smart Bio-devices, National Yang Ming Chiao Tung University, Hsinchu, Taiwanand; Department of Biological Science and Technology, National Yang Ming Chiao Tung University, Hsinchu, Taiwan; Division of Pulmonary and Critical Care Medicine, Kaohsiung Medical University Hospital, Kaohsiung, Taiwan; Department of Internal Medicine, Kaohsiung Medical University Hospital, Kaohsiung, Taiwan; Division of Pulmonary Medicine and Pulmonary Research Center, Wanfang Hospital, Taipei Medical University, Taipei, Taiwan; Department of Biological Science and Technology, National Yang Ming Chiao Tung University, Hsinchu, Taiwan

**Keywords:** interpretable machine learning, latent tuberculosis infection, rifapentine, systemic drug reaction, transcriptome

## Abstract

**Background:**

Systemic drug reaction (SDR) is a major safety concern with weekly rifapentine plus isoniazid for 12 doses (3HP) for latent tuberculosis infection (LTBI). Identifying SDR predictors and at-risk participants before treatment can improve cost-effectiveness of the LTBI program.

**Methods:**

We prospectively recruited 187 cases receiving 3HP (44 SDRs and 143 non-SDRs). A pilot cohort (8 SDRs and 12 non-SDRs) was selected for generating whole-blood transcriptomic data. By incorporating the hierarchical system biology model and therapy–biomarker pathway approach, candidate genes were selected and evaluated using reverse-transcription quantitative polymerase chain reaction (RT-qPCR). Then, interpretable machine learning models presenting as SHapley Additive exPlanations (SHAP) values were applied for SDR risk prediction. Finally, an independent cohort was used to evaluate the performance of these predictive models.

**Results:**

Based on the whole-blood transcriptomic profile of the pilot cohort and the RT-qPCR results of 2 SDR and 3 non-SDR samples in the training cohort, 6 genes were selected. According to SHAP values for model construction and validation, a 3-gene model for SDR risk prediction achieved a sensitivity and specificity of 0.972 and 0.947, respectively, under a universal cutoff value for the joint of the training (28 SDRs and 104 non-SDRs) and testing (8 SDRs and 27 non-SDRs) cohorts. It also worked well across different subgroups.

**Conclusions:**

The prediction model for 3HP-related SDRs serves as a guide for establishing a safe and personalized regimen to foster the implementation of an LTBI program. Additionally, it provides a potential translational value for future studies on drug-related hypersensitivity.

## Teaser

By integrating clinical samples, bioinformatic techniques, and explainable machine learning, we provide a blood-based prediction model to foresee rifapentine-based treatment–related systemic drug reaction, leading to increased public acceptance of public health policy on latent tuberculosis infection intervention.

Treatment for latent tuberculosis infection (LTBI) is crucial for tuberculosis (TB) elimination [1]. A short-course regimen consisting of once-weekly high-dose rifapentine plus isoniazid for a total of 12 doses, termed 3HP, is recommended for adults and children aged ≥2 years for its similar efficacy, lower hepatotoxicity, and higher completion rate compared with 6- or 9-month isoniazid monotherapy [2]. However, unpredictable systemic drug reaction (SDR) occurs in 3.5%–11.2% of participants who receive the 3HP regimen, accounting for 38%–50% of permanent discontinuation of 3HP treatment [3]. Among those who experience SDRs, 0.6%–4.2% may require hospitalization [3-6].

The associated phenotypic risk factors for 3HP-related SDRs include female sex [4], age >35 years (particularly between 35 and 65 years) [3, 4], and low body mass index (BMI) [4]. The mechanisms of these hypersensitivity reactions remain unclear and are likely to be multifactorial, involving direct toxicity of the drug or its metabolites, host immunity constitution, formation of the circulating antibody–antigen complex [7], plasma isoniazid concentration, and genetics [8, 9]. For precision medicine against LTBI, a comprehensive phenotypic anchoring of genome-wide gene-expression signatures is necessary [10]. This will enable the establishment of a prediction model for SDRS to provide solid evidence for related pathogenesis and a decision aid for TB preventive therapy and to facilitate cost-effective implementation of an LTBI program by using the 3HP regimen.

Some studies have proposed gene signatures for LTBI and TB diagnosis [11-13]. However, these studies have not provided absolute thresholds of gene signatures, and the transcriptome-based prediction of SDRs from blood samples before 3HP treatment is lacking. To address these issues, we developed a hierarchical system biology model (HiSBiM) [14] and therapy–biomarker pathway approach to identify a transcriptional signature for 3HP-related SDRs in peripheral blood and then constructed interpretable SDR prediction models with a universal cutoff value by using SHapley Additive exPlanations (SHAP) values [15] to foresee 3HP-related SDRs in LTBI individuals before 3HP treatment.

## METHODS

### Study Design

Between January 2017 and January 2020, 447 participants who ever took at least 1 dose of 3HP in 2 medical centers were enrolled. Among them, 44 developed SDRs during 3HP treatment. All of the 44 SDR cases and 143 of the 404 non-SDR cases were selected to identify SDR-related biomarkers. The 8 SDR and 12 non-SDR cases enrolled between January 2017 and June 2019 were grouped as the pilot cohort. The remaining cases, enrolled after July 2019, were randomly divided 80% into the training cohort (28 SDR and 104 non-SDR cases) and 20% into the testing cohort (8 SDR and 27 non-SDR cases).

**Figure 1.**
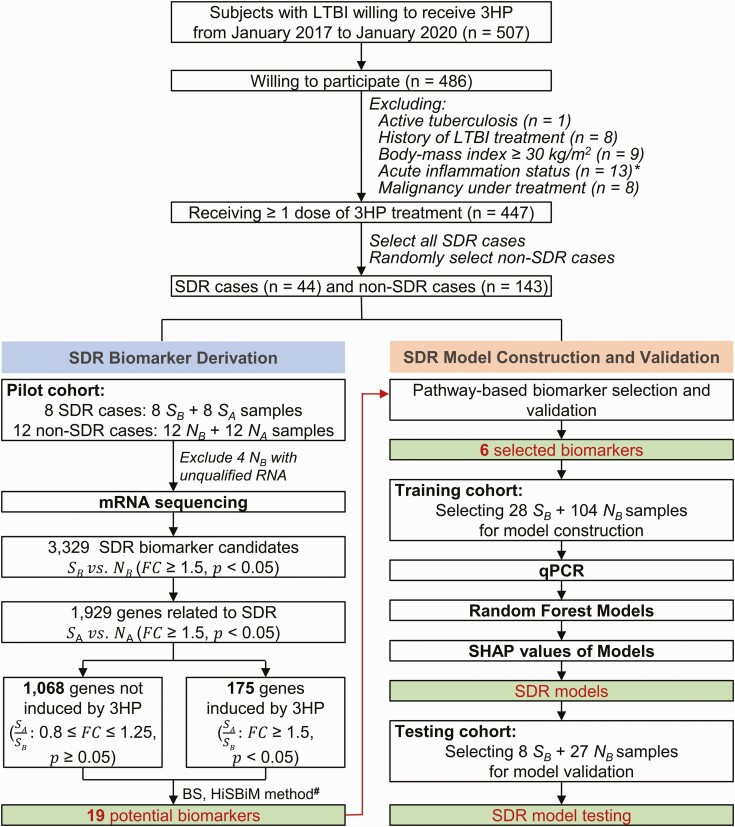
Overview of the case enrollment and analysis plan. The main steps included case enrollment, biomarker derivation, SDR model construction, and validation. *S*_*A*_ represents samples from the SDR group after 3HP treatment; *S*_*B*_ represents samples from the SDR group before 3HP treatment; *N*_*A*_ represents samples from the non-SDR group after 3HP treatment; and *N*_*B*_ represents samples from the non-SDR group before 3HP treatment. ^∗^Seven cases had acute upper respiratory infection, 2 had urinary tract infection, 2 had influenza, 1 had pneumonia, and 1 had cellulitis. ^#^Please see Supplementary Methods. Abbreviations: 3HP, weekly rifapentine plus isoniazid for 12 doses; BS, biological similarity score; HiSBiM, hierarchical system biology model; LTBI, latent tuberculosis infection; qPCR, quantitative polymerase chain reaction; SDR, systemic drug reaction; SHAP, SHapley Additive exPlanations.

The study consisted of 4 main parts (Figure 1). First, the pilot cohort was used to identify candidate transcripts that showed a high association with SDR occurrence from peripheral blood transcriptomic profiles through the incorporation of biological similarity score (BS) and HiSBiM (Supplementary Methods). Second, 6 transcripts were selected based on the candidate gene expression profiles by using reverse-transcription quantitative polymerase chain reaction (RT-qPCR; Supplementary Methods, Supplementary Table 1) of pre-3HP samples after pathway-based biomarker selection and validation from 3 SDR and 2 non-SDR samples in the training cohort. Third, random forest (RF) models and SHAP values were applied for SDR predictive model construction by using all samples in the training cohort. Finally, the accuracy of the SDR predictive model was independently evaluated using the testing cohort.

### Participant Selection Criteria

Individuals were eligible for enrollment if they were aged ≥13 years and received ≥1 dose of 3HP treatment for LTBI, which was diagnosed using QuantiFERON-TB Gold In-tube (Cellestis/Qiagen, Carnegie, Australia). Participants with active TB, a history of LTBI treatment, obesity (BMI >30 kg/m^2^, which inevitably resulted in a low dosage of isoniazid and rifapentine), malignancy under treatment, living with human immunodeficiency virus, and acute illness with inflammatory symptoms and signs were excluded.

### Study Protocols and SDR Assessment

Eligible participants received 12 doses of weight-adjusted weekly high-dose rifapentine plus isoniazid under supervision (Supplementary Methods). Within 2 days after each dose of 3HP, the manifestations [4] and severity [16] of any adverse drug reaction (ADR) were assessed and recorded. Blood samples were collected before 3HP treatment, monthly after 3HP treatment, and after SDR development (Supplementary Methods).

### Definition of SDR

SDR phenotypes included hypotension (systolic blood pressure <90 mm Hg), urticaria, angioedema, acute bronchospasm, or conjunctivitis and more than 4 of the following symptoms occurring concurrently (more than 1 of which had to be grade ≥2): weakness, fatigue, nausea, vomiting, headache, fever, aches, sweating, dizziness, shortness of breath, flushing, or chills [4]. The causative association of 3HP was determined using the Naranjo algorithm [17].

### Bioinformatics Analysis and Candidate Gene Selection

**Figure 2.**
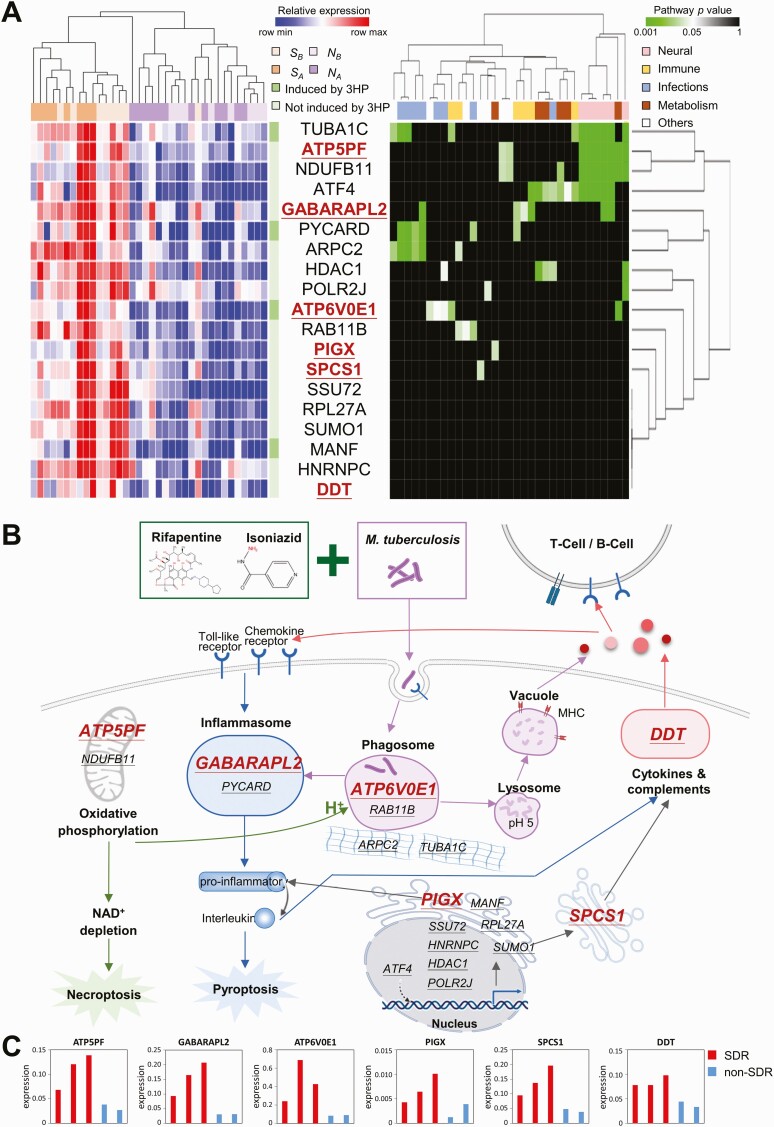
Gene expression signature and therapy–biomarker pathway for predicting SDR in participants with latent tuberculosis infection before treatment with 3HP. *A,* Heat map and hierarchical clustering of gene expression (left) and pathway (right) for 19 potential biomarkers in *S*_*A*_, *S*_*B*_, *N*_*A*_, and *N*_*B*_ samples. Of the 19 genes, 4 are significantly upregulated after 3HP treatment (dark green), whereas the other 15 genes are not (light green). Among these genes, the 6 selected potential biomarkers are marked in red. *B,* Therapy–biomarker pathways for illustrating potential genes associated with SDR development under 3HP treatment. For better visualization, the 19 potential biomarkers are underlined with the 6 selected genes marked in red. *C,* Bar chart for the expression of *ATP5PF*, *GABARAPL2*, *ATP6V0E1*, *PIGX*, *SPCS1*, and *DDT* in 3 SDR (red) and 2 non-SDR (blue) samples collected before 3HP treatment. The expression levels were validated through reverse-transcription quantitative polymerase chain reaction. Abbreviations: 3HP, weekly rifapentine plus isoniazid for 12 doses; MHC, major histocompatibility complex; *N*_*A*_, samples from the non-SDR group after 3HP treatment; *N*_*B*_, samples from the non-SDR group before 3HP treatment; NAD, nicotinamide adenine dinucleotide; SDR, systemic drug reaction; *S*_*A*_, samples from the SDR group after 3HP treatment; *S*_*B*_, samples from the SDR group before 3HP treatment.

For RNA-seq data (GSE174552) obtained from the pilot cohort [18], we used the HisBiM and therapy-biomarker pathway approach to identify transcriptomic signatures (Figure 2). A fold change of ≥1.5 and *P* < .05 (*t* test) were set to identify 1243 differentially expressed genes (DEGs) before and after 3HP treatment (Figure 1). To investigate the composition of cell types of each sample, we used CIBERSORT to analyze the signatures of the DEGs [19].

To infer potential biomarkers, we first analyzed enrichment pathways using hypergeometric distribution of these 1243 DEGs. Then, we proposed an integrated gene score (S_IG_) to calculate the importance of each DEG (Supplementary Methods). Third, these DEGs were clustered into 60 groups based on BS by using hierarchical clustering analysis (Supplementary Methods) [20]. We then identified 19 potential biomarkers by integrating BS and S_IG_ scores and applied RT-qPCR for further validation (Figure 2A). Finally, 6 genes were determined using the therapy–biomarker pathway approach and domain knowledge (Figure 2B).

### Interpretable Prediction Model

To build a robust prediction model with a universal cutoff value, the training cohort was used to develop interpretable RF models with SHAP values (Supplementary Methods). First, to avoid overfitting in the prediction of 3HP-related SDRs, we systematically tested parameters (eg, trees, features, samples, and leaf nodes) to investigate the characteristics of RF models through the scikit-learn Python library (*sklearn.ensemble.RandomForestClassifier*) [21].

Next, the SHAP method was applied to these models to reveal the impact of each gene on each sample. The default values were then used to build simple and robust models. The sum of SHAP values of a sample’s input features was used to compare universal cutoff values for predicting 3HP-related SDRs. Finally, the independent testing cohort was used to evaluate the performance of the models based on sensitivity (sen), specificity (spe), and their geometric mean (G-mean, calculated as $\sqrt{sen\times spe}$)  [22].

### Sensitivity and Statistical Analyses

We assessed the performance of the selected SDR predictive models in the joint population of training and testing cohorts as well as various subpopulations, stratified by age, sex, BMI, presence of systemic comorbidity, and renal function (using estimated glomerular filtration rate [23] as a surrogate).

The demographic data, comorbidity status, laboratory data, treatment course, and all ADRs were programmatically collected. The intergroup difference was analyzed using the Mann–Whitney *U* test for continuous variables and the *χ*^2^ test for categorical variables. Statistical significance was set at 2-sided *P* < .05. The exact binomial method was applied to calculate 95% confidence intervals (CIs) of sensitivity and specificity. Standard normal distribution was used to calculate the 95% CI of the area under the receiver operating characteristic curve.

### Ethic Approval

The study was approved by the institutional ethics committees of National Taiwan University Hospital and Kaohsiung Medical University Hospital. Each participant provided informed consent before enrollment.

## RESULTS

### Selection of Study Participants

Among the 44 SDR cases and 143 non-SDR cases (Figure 1), some had been reported in previous studies (Supplementary Results). Of the 44 SDR cases, 38 (86.3%) presented as flu-like syndrome (Supplementary Table 2), and 93.2% had ≥1 flu-like symptoms. Most SDRs occurred 4–5 hours after the third dose of 3HP and persisted for a median duration of 18–29 hours. The occurrence of SDRs resulted in permanent discontinuation of 3HP in 13 (30%) SDR cases.

The baseline characteristics of the SDR and non-SDR participants were similar, except that the SDR group in the training cohort had a lower prevalence of hypertension and diabetes mellitus and a lower level of serum alanine transaminase (Table 1; Supplementary Table 3).

**Table 1.** Clinical Characteristics of Participants

Characteristic	Pilot Cohort		Training Cohort		Testing Cohort	
	SDR (n = 8)	non-SDR (n = 12)	SDR (n = 28)	non-SDR (n = 104)	SDR (n = 8)	non-SDR (n = 27)
Age, y	51.2 ± 10.8	48.8 ± 10.8	47.7 ± 14.1	50.8 ± 19.8	48.1 ± 14.0	55.1 ± 21.3
≤35	1 (12.5%)	1 (8.3%)	6 (21.4%)	30 (28.8%)	2 (25.0%)	5 (18.5%)
>35	7 (87.5%)	11 (91.7%)	22 (78.6%)	74 (71.2%)	6 (75.0%)	22 (81.5%)
Female sex	6 (75.0%)	10 (83.3%)	17 (60.7%)	49 (47.1%)	4 (50.0%)	12 (44.4%)
Body mass index, kg/m^2^	22.6 ± 2.7	23.3 ± 2.5	23.7 ± 3.3	24.7 ± 3.9	24.0 ± 3.6	25.3 ± 4.6
Diabetes mellitus	1 (12.5%)	2 (16.7%)	2 (7.1%)	25 (24.0%)^a^	1 (12.5%)	8 (29.6%)
Hypertension	1 (12.5%)	1 (8.3%)	4(14.3%)	36 (34.6%)^a^	1 (12.5%)	11 (40.7%)
Autoimmune^b^	1 (12.5%)	0	1 (3.6%)	2 (1.9%)	0	0
Isoniazid/Rifapentine dose, mg/kg	15.1 ± 1.8	15.2 ± 1.5	14.4 ± 2.6	13.6 ± 2.2	14.8 ± 2.3	13.4 ± 2.2
Hemoglobin, g/dL	13.2 ± 1.3	13.9 ± 1.1	14.0 ± 1.6	14.1 ± 1.8	13.8 ± 1.4	14.1 ± 1.7
Leukocyte, K/µL	6.6 ± 1.2	7.2 ± 1.3	6.5 ± 1.3	7.2 ± 1.8^a^	6.9 ± 1.4	7.9 ± 2.5
Platelet, K/µL	270 ± 36	281 ± 60	286 ± 61	267 ± 62	276 ± 76	259 ± 72
Alanine transaminase, U/L	22.0 ± 12.2	18.0 ± 11.3	19.6 ± 8.2	24.2 ± 14.8^a^	24.4 ± 17.2	20.0 ± 5.8
Estimated glomerular filtration rate, mL/min/1.73m^2^	105 ± 30	112 ± 30	102 ± 37	95 ± 25	99 ± 18	86 ± 31
QuantiFERON response, IU/mL^c^	2.1 ± 2.1	1.9 ± 2.5	1.8 ± 1.6	2.4 ± 25	2.3 ± 3.2	3.6 ± 2.8
Any adverse event due to weekly rifapentine plus isoniazid for 12 doses^d^	8 (100.0%)	8 (66.7%)	28(100.0%)	63 (60.6%)	8 (100.0%)	12 (44.4%)
Grade 3	2 (25.0%)	1 (8.3%)	3 (10.7%)	1 (1.0%)	2 (25.0%)	0
Grade 2	6 (75.0%)	1 (8.3%)	25 (89.3%)	22 (21.2%)	6 (75.0%)	3 (11.1%)
Grade 1	0	6 (50.0%)	0	40 (38.5%)	0	9 (33.3%)

Data are presented as number (percentage) or mean ± standard deviation.

Abbreviation: SDR, systemic drug reaction.

*P* < .05 between SDR and non-SDR groups.

The autoimmune disease was rheumatoid arthritis for the pilot cohort and 2 non-SDR cases in the training cohort. The autoimmune disease was Sjögren syndrome for the SDR case in the training cohort.

QuantiFERON response was defined as the difference between the interferon-gamma levels of Antigen and Nil tubes as obtained using the QuantiFERON-TB Gold in-Tube test (Cellestis/Qiagen, Carnegie, Australia).

For detailed information on each adverse event, please see Supplementary Table 3.

### Selection of Candidate Transcripts

Because of unqualified RNA, 4 non-SDR samples collected before treatment were excluded from mRNA sequencing (Figure 1). On the basis of S_IG_ and BS scores, we identified 19 candidate genes with significantly higher expression in the 16 SDR samples (orange) than in the 20 non-SDR samples (purple; Figure 2A, left). The accuracy in the discrimination of SDR and non-SDR populations was 94% (34 of 36).

By analyzing the hypergeometric distribution, 4 genes (ie, *ATP5PF*, *ATF4*, *NDUFB11*, and *TUBA1C*) were involved in the neural-related pathways in Kyoto Encyclopedia of Genes and Genomes (*P* < .05; Figure 2A, right; Supplementary Figure 1). Other genes were involved in immune, infection, and metabolism-related pathways, implying the hyperinflammatory status. Furthermore, the therapy–biomarker pathway was constructed based on above 19 genes to illustrate and classified into 6 biological functions (Figure 2B; Supplementary Results).

The RT-qPCR results of 3 SDR and 2 non-SDR samples (Supplementary Table 4) in the training cohort revealed that 11 of the 19 genes are consistently highly expressed in SDR samples (Figure 2C; Supplementary Figure 2). According to the therapy–biomarker pathway approach and RT-qPCR results of these 5 samples, 6 potential biomarkers, namely, *ATP5PF*, *ATP6V0E1*, *PIGX*, *SPCS1*, *GABARAPL2*, and *DDT*, were further selected (Supplementary Table 5).

According to CIBERSORT analysis, a high proportion of activated dendritic cells (11.2% vs 2.6%, *P* = .006) and a low proportion of monocytes (3.7% vs 14.3%, *P* = .009) before 3HP treatment were significantly associated with SDRs (Supplementary Figure 3).

### SDR Prediction Models

**Figure 3.**
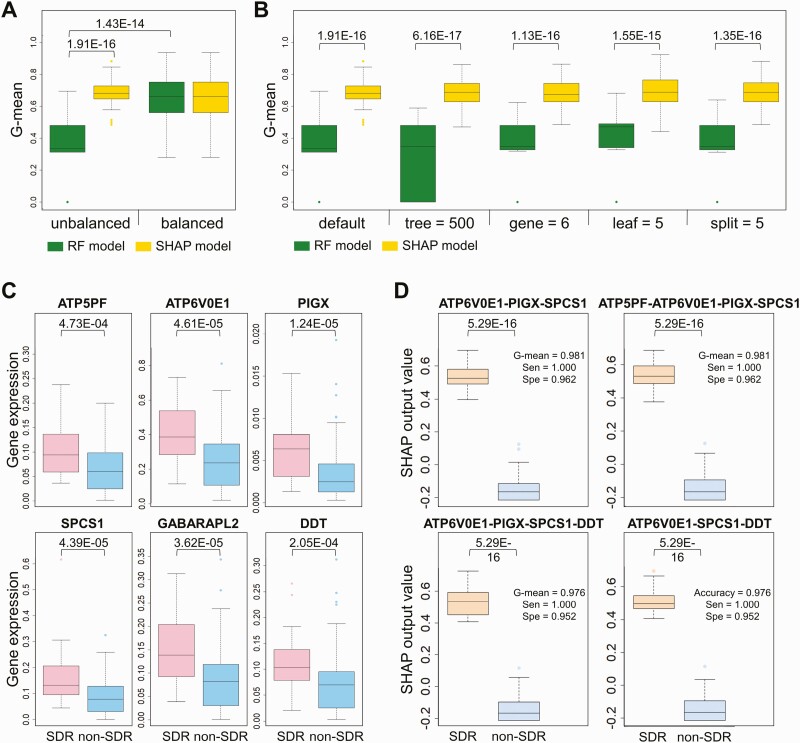
RF model and SHAP models of the 6 selected genes to discriminate pretreatment samples collected from participants with and without SDR in the training cohort. *A,* Box plot of the G-mean of sensitivity and specificity of the RF model (green) and SHAP model (yellow) in 50 random unbalanced testing sets (8 SDR and 27 non-SDR samples) and balanced testing sets (8 SDR and 8 non-SDR samples). The *P* values were calculated using the Mann–Whitney *U* test. *B,* Box plot of the G-mean of the RF model (green) and SHAP model (yellow) in 50 random testing sets (8 SDR and 27 non-SDR samples) under various model parameters, including default, number of trees (500), number of genes (6), minimum number of samples required to be at a leaf node (leaf = 5), and minimum number of samples required to split an internal node (split = 5). The *P* values were calculated using the Mann–Whitney *U* test. *C,* Box plot of the expressions of *ATP5PF*, *ATP6V0E1*, *PIGX*, *SPCS1*, *GABARAPL2*, and *DDT* for the 28 SDR (pink) and 104 non-SDR (blue) training samples. Boxes indicate the sample median and interquartile range, whereas bars and colored dots indicate the range and outliers, respectively. Data were analyzed using the Mann–Whitney *U* test. *D,* Box plot of the SHAP output values of the 4 best performing models in SDR (orange) and non-SDR (steel blue) training samples. Abbreviations: G-mean, geometric mean; RF, random forest; SDR, systemic drug reaction; Sen, sensitivity; SHAP, SHapley Additive exPlanations; Spe, specificity.

To establish a robust prediction model with a universal cutoff value, we developed interpretable RF models with SHAP. Comparisons between RF and SHAP models can be summarized as follows: first, the SHAP model (yellow) has similar performance, with a G-mean of approximately 0.7 for unbalanced and balanced datasets (Figure 3A). Conversely, the G-mean of the RF model for the unbalanced dataset (green) was significantly lower than the G-means of the RF models for balanced datasets and the G-means of the SHAP models for unbalanced datasets (*P* < .001). Second, SHAP models significantly outperformed RF models in various parameter settings, including default, 500 trees, 6 genes, 5 samples required to be at a leaf node, and 5 samples required to split an internal node (*P* < .001; Figure 3B). On the basis of these results, we used the SHAP model to provide a universal cutoff value and foresee 3HP-related SDRs before preventive therapy.

The expression profiles by RT-qPCR of all 6 genes were significantly higher in the SDR group compared with the non-SDR group in the training cohort (Figure 3C). Systemic combinations of the 6 selected gene expression signatures were used to build SHAP models. Among the 14 top-ranked models, the worst G-mean in the training cohort was 0.976, whereas the best G-mean was 0.985 (Supplementary Table 6).

Gene clustering analysis showed that the gene expressions of *ATP5PF* and *DDT* were similar in the 132 training samples. Furthermore, *SPCS1* and *GABARAPL2* had a similar expression level (Supplementary Figure 4). On the basis of the findings of redundancy, we selected 4 models with a high G-mean, shown in Figure 3D (details in Supplementary Table 6) for further analysis of SDR prediction. The box plots show significant differences in the SHAP output value between SDR and non-SDR samples (*P* < .001; Figure 3D). Under a universal cutoff, the SHAP model provided an output value for each sample in these 4 selected models (Supplementary Figure 5).

### Testing of SDR Prediction Models

**Figure 4.**
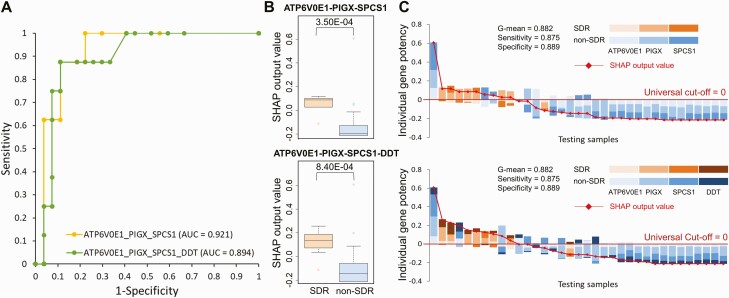
SHAP models of the 2 selected models to discriminate pretreatment samples collected from participants with and without SDR in the testing cohort. *A,* Receiver operating characteristic curve and AUC of the 3-gene (ATP6V0E1-PIGX-SPCS1) and 4-gene (ATP6V0E1-PIGX-SPCS1-DDT) models in the testing cohort. *B,* Box plot of the SHAP output values of 2 models for the 8 SDR (orange) and 27 non-SDR (steel blue) testing samples. Boxes indicate median and interquartile range, whereas bars and colored dots indicate the range and outliers, respectively. Data were analyzed using the Mann–Whitney *U* test. *C,* Interpretation of SDR predictive models with universal cutoffs for SDR (orange) and non-SDR (blue) testing samples. The SHAP output value in each sample is a red diamond, and the universal cutoff is 0 (deep red line). G-mean represents the geometric mean of sensitivity and specificity. Abbreviations: AUC, area under the curve; SDR, systemic drug reaction; SHAP, SHapley Additive exPlanations.

The expression profiles based on RT-qPCR of all 6 selected genes were significantly higher in the SDR group compared with the non-SDR group in the testing cohort (*P* < .005; Supplementary Figure 6). The performance of the 4 selected SDR prediction models for the testing samples is shown in Supplementary Table 7. The area under the curve of the 2 best prediction models, that is, ATP6V0E1-PIGX-SPCS1 (yellow) and ATP6V0E1-PIGX-SPCS1-DDT (green), were 0.921 and 0.894, respectively (Figure 4A). The SHAP values of SDR samples were significantly higher than those of non-SDR samples (*P* < .001; Figure 4B). The interpretation models provide the SHAP output value (Figure 4C), and *PIGX* is often the most influential in prediction.

### SDR Prediction Model in Subpopulations

**Figure 5.**
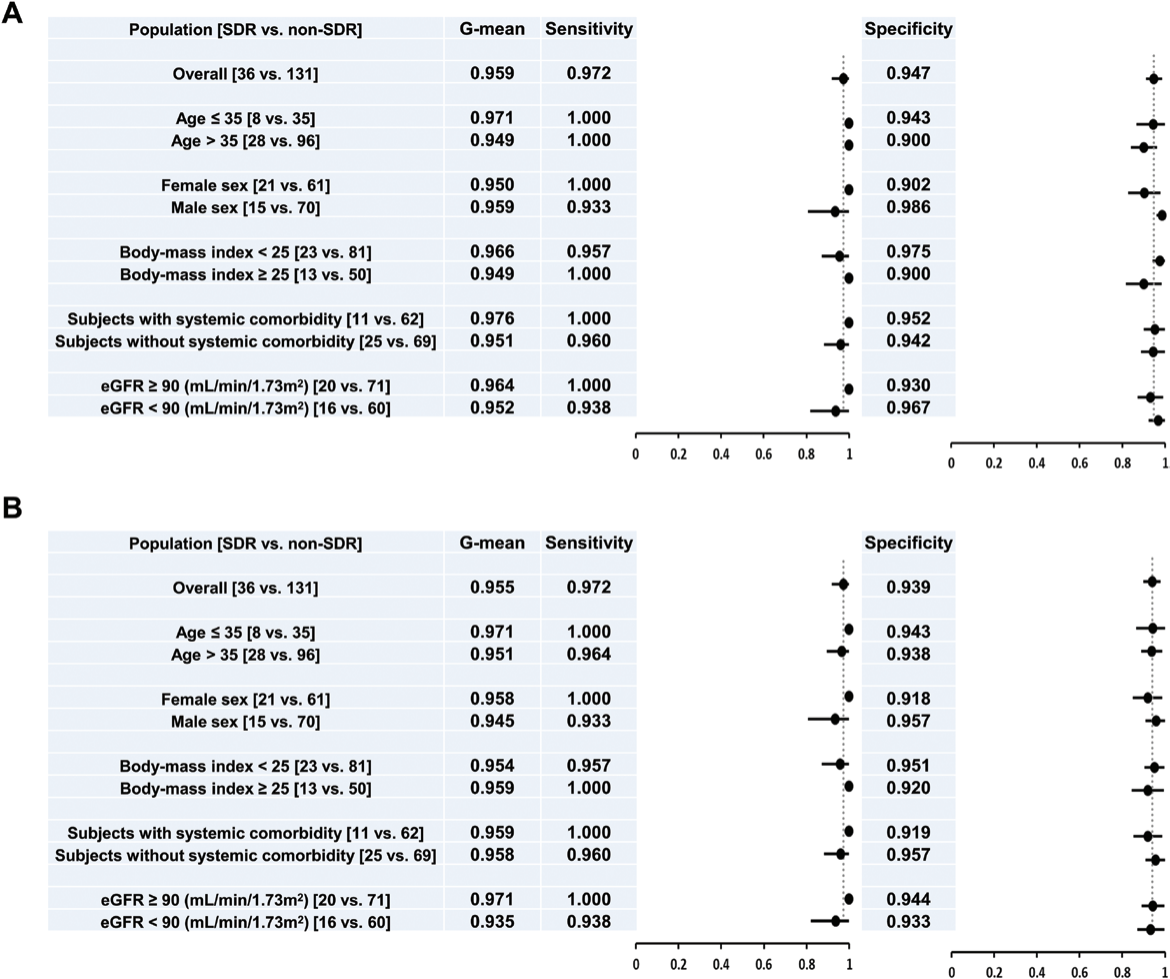
Forest plots of the performance of ATP6V0E1-PIGX-SPCS1 *(A)* and ATP6V0E1-PIGX-SPCS1-DDT *(B)* models used to predict SDR from pretreatment samples in the joint population of training and testing cohorts as well as various subgroups. Abbreviations: eGFR, estimated glomerular filtration rate; SDR, systemic drug reaction.

The best SDR prediction models ATP6V0E1-PIGX-SPCS1 and ATP6V0E1-PIGX-SPCS1-DDT for the joint population from training and testing cohorts had G-means of 0.959 (sen = 0.972, spe = 0.947; Figure 5A) and 0.955 (sen = 0.972, spe = 0.939; Figure 5B), respectively, by using 0 as the cutoff value for SHAP output values. The performance of these SDR prediction models was similar across different subpopulations (sen = 0.933–1.000, spe = 0.900–0.986).

## DISCUSSION

This is the first study to provide an accurate prediction model with a universal cutoff value through the integration of clinical samples, bioinformatic techniques, and explainable machine learning to foresee SDR occurrence before 3HP treatment for LTBI. In the management of 3HP-related SDRs, in addition to treatment and supportive care, we herein propose a practical module that consists of the expression profiles of 3–4 genes by using peripheral blood samples to provide a decision aid for safely selecting people with LTBI to undergo 3HP, the most convenient regimen for TB prevention. The SDR prediction model represents a major step forward for precision medicine in TB preventive therapy. As the ancient Greek physician Hippocrates said, “the first and most important oath for being a doctor is do no harm.” This is especially true in the field of preventive medicine. Using the SDR prediction model invented in the current study, almost 97.2% of 3HP-related SDRs can be foreseen and prevented. The intervention program for LTBI treatment could therefore be successfully, safely, and cost-effectively rolled out.

An SDR usually occurred 4–5 hours after the third 3HP dose, most likely due to immune response to certain drug acting as an allergen to elicit endogenous proteins or peptides [24]. Results of CIBERSORT analysis suggest that the difference in immune cell constitution before treatment may be an important driver for T cell–mediated hypersensitivity reaction against 3HP. The proliferation of circulating activated dendritic cells at baseline may result in the augmentation of immune response with reexposure to the same antigen [25].

SDR prediction models are based on interpretable SHAP models rather than RF models because of 3 advantages (Figure 3A and 3B). First, the SHAP model is suitable for unbalanced data without the need for adjusting any model parameters. Second, under various machine learning parameters, the SHAP model is more stable and accurate than the RF model. Last, the SHAP model can provide a universal cutoff value based on the comprehensive consideration of the contribution of every feature in each sample.

The underlying pathogenetic mechanism of the 3HP-related hypersensitivity reactions remains unclear. *PIGX*, a type I transmembrane protein in the endoplasmic reticulum, is required for the complete activation of the regulatory and effector functions of T lymphocytes [26]. *GABARAPL2* [27, 28] and *ATP6V0E1* [29] both encode proteins essential for autophagy in macrophages. *DDT* (D-dopachrome tautomerase) regulates a diverse range of physiological functions related to innate immunity and inflammation [30]. *SPCS1* (signal peptidase complex subunit 1) is involved in the post-translational processing of the structural proteins of Japanese encephalitis virus, Zika virus, and hepatitis C virus [31, 32]. *ATP5PF* encodes ATP synthase-coupling factor 6 and enhances oxidative phosphorylation in mitochondria during inflammation [33]. However, the exact physiological functions and molecular interactions of the aforementioned genes on 3HP-related SDRs remain undetermined.

The current study has 3 unique characteristics. First, we used a rather large number of clinical samples with clear clinical phenotypes [3]. Second, we proposed a therapy–biomarker pathway approach to identify candidate signatures and developed interpretable machine learning models with SHAP values to provide a universal cutoff value for SDR prediction. Third, the accuracy of the SDR prediction model constructed in the training cohort was confirmed to be high by using an independent testing cohort under a standardized framework of validation. We believe that the multidisciplinary collaboration and the way the clinical problem was addressed as well as the way the SDR prediction model was constructed and tested could be a research model for other severe ADRs in the future.

The current study has some limitations. First, SDRs are likely to be heterogenous. The exact mechanisms that surround both the predictive transcriptomes and the intercellular processing of host immunity remain unexplained. Second, we cannot identify the offending drug for SDRs. However, the finding that 4 of the 19 potential genes identified in the pilot cohort were involved in the neural-related pathways and the well-documented neurotoxicity of isoniazid (INH) [34] suggest that further investigation of the potential contribution of INH to SDR is necessary. Third, this study only recruited Asians. External validation using non-Asian populations is necessary to confirm the performance of the current SDR prediction models. Fourth, the best signatures depended on the PIGX transcript, yet it was present in tiny amounts. However, it may not be a critical limitation in clinical application given that the average cycle threshold value was 30.3 ± 2.8 and 31.2 ± 1.9 in SDR and non-SDR groups, respectively, suggesting that the low expression level of *PIGX* in most clinical samples should remain detectable by RT-qPCR. Last, the current predictive tool would likely be unaffordable or difficult to logistically implement in resource-limited countries, which have the highest TB burden. Further optimization and simplification of the technology or expanding our evaluation to assess its utility in whole blood, as others have similarly explored, might make this tool more feasible to implement.

## CONCLUSIONS

By using RNA sequencing to create a global picture of cellular function across all expressed genes, bioinformatic tools to select candidate genes, and machine learning to interpret RT-qPCR results, we provided SDR prediction models with a universal cutoff value to foresee 3HP-related SDRs and provide an aid for treatment decisions before preventive therapy. The SDR prediction model represents a major step forward for precision medicine in TB preventive therapy and may speed up the global uptake of public health programs against LTBI for TB elimination.

## Supplementary Data

Supplementary materials are available at *Clinical Infectious Diseases* online. Consisting of data provided by the authors to benefit the reader, the posted materials are not copyedited and are the sole responsibility of the authors, so questions or comments should be addressed to the corresponding author.

ciac003_suppl_Supplementary_Material

## Notes

***Acknowledgments.*** The authors thank the staff of the Eighth Core Lab, Department of Medical Research at the National Taiwan University Hospital for their technical support.

Deidentified individual participant data that underlie the results reported in this article will be available immediately following publication upon request to the corresponding author.

***Author Contributions*.** Manuscript writing and figure: H. L. H., J. Y. L., Y. S. L., J. Y. W., J. M. Y.; study design: H. L. H., C. H. L., J. Y. W., J. M. Y., I. W. C.; data collection: H. L. H., M. R. L., C. H. L., J. Y. W.; data analysis and interpretation: H. L. H., J. Y. L., Y. S. L., I. H. L., S. H. H., Y. W. H., M. R. L., C. H. L., J. Y. W., J. M. Y., I. W. C.; manuscript revision: J. Y. W., J. M. Y., I. W. C.

***Disclaimer.*** The funders had no role in the study design, data collection and analysis, decision to publish, or preparation of the manuscript.

***Financial support.*** The work was supported by grants from the Ministry of Health and Welfare (MOHW106-CDC-C-11400104, MOHW107-CDC-C-114-000105, and MOHW108-CDC-C-114-000108), Ministry of Science and Technology (MOST107-2314-B-037-106-MY3, MOST108-2314-B-002-190-MY3, and MOST109-2314-B-037-085-MY3), Kaohsiung Municipal Ta-Tung Hospital (KMTTH-109-R001, KMTTH-110-R002), Kaohsiung Medical University (KMUH106-6M07), the National Chiao Tung University–Kaohsiung Medical University Joint Research Project (NCTUKMU109-AI-02), the MOST Joint Research Center for AI Technology and All Vista Healthcare (MOST109-2634-F-009-021 and MOST107-2634-F-002-019), National Health Research Institutes (NHRI-EX109-10504PI and NHRI-EX111-11017BI), Smart Platform of Dynamic Systems Biology for Therapeutic Development project, Center for Intelligent Drug Systems and Smart Bio-devices, Center for Bioinformatics Research by the Ministry of Education ATU program, and National Yang Ming Chiao Tung University.

## References

[1] Pai M , Behr MA, Dowdy D, et al Tuberculosis. Nat Rev Dis Primers 2016; 2:16076. 27784885 10.1038/nrdp.2016.76

[2] World Health Organization. WHO Guidelines Approved by the Guidelines Review Committee. WHO consolidated guidelines on tuberculosis: tuberculosis preventive treatment: module 1: prevention. Geneva, Switzerland: World Health Organization, 2020. https://www.who.int/publications/i/item/9789240001503. Accessed 15 December 2021.

[3] Huang HL , Lee MR, Cheng MH, et al Impact of age on outcome of rifapentine-based weekly therapy for latent tuberculosis infection. Clin Infect Dis 2021; 73:e1064–71. 33215187 10.1093/cid/ciaa1741 PMC8423464

[4] Sterling TR , Moro RN, Borisov AS, et al Flu-like and other systemic drug reactions among persons receiving weekly rifapentine plus isoniazid or daily isoniazid for treatment of latent tuberculosis infection in the PREVENT tuberculosis study. Clin Infect Dis 2015; 61:527–35. 25904367 10.1093/cid/civ323 PMC4560029

[5] Walker RE , Bass S, Srinivas P, Miranda C, Johnson L, Pallotta AM. Evaluation of 3 months of once-weekly rifapentine and isoniazid for latent tuberculosis infection. Ann Pharmacother 2020; 54:457–63. 31729245 10.1177/1060028019888855

[6] Schmit KM , Wortham JM, Ho CS, Powell KM. Analysis of severe adverse events reported among patients receiving isoniazid-rifapentine treatment for latent *Mycobacterium tuberculosis* infection—United States, 2012-2016. Clin Infect Dis 2020; 71:2502–5. 10.1093/cid/ciaa286 PMC8020883 32185390

[7] Brooks KM , George JM, Pau AK, et al Cytokine-mediated systemic adverse drug reactions in a drug-drug interaction study of dolutegravir with once-weekly isoniazid and rifapentine. Clin Infect Dis 2018; 67:193–201. 29415190 10.1093/cid/ciy082 PMC6248641

[8] Lee MR , Huang HL, Lin SW, et al Isoniazid concentration and NAT2 genotype predict risk of systemic drug reactions during 3HP for LTBI. J Clin Med 2019; 8:812. 10.3390/jcm8060812 PMC6616849 31174321

[9] Yu YY , Tsao SM, Yang WT, et al Association of drug metabolic enzyme genetic polymorphisms and adverse drug reactions in patients receiving rifapentine and isoniazid therapy for latent tuberculosis. Int J Environ Res Public Health 2019; 17:210. 10.3390/ijerph17010210 PMC6981901 31892222

[10] Byron SA , Van Keuren-Jensen KR, Engelthaler DM, Carpten JD, Craig DW. Translating RNA sequencing into clinical diagnostics: opportunities and challenges. Nat Rev Genet 2016; 17:257–71. 26996076 10.1038/nrg.2016.10 PMC7097555

[11] Anderson ST , Kaforou M, Brent AJ, et al Diagnosis of childhood tuberculosis and host RNA expression in Africa. N Engl J Med 2014; 370:1712–23. 24785206 10.1056/NEJMoa1303657 PMC4069985

[12] Berry MP , Graham CM, McNab FW, et al An interferon-inducible neutrophil-driven blood transcriptional signature in human tuberculosis. Nature 2010; 466(7309): 973–977. 20725040 10.1038/nature09247 PMC3492754

[13] Sweeney TE , Braviak L, Tato CM, Khatri P. Genome-wide expression for diagnosis of pulmonary tuberculosis: a multicohort analysis. Lancet Respir Med 2016; 4:213–24. 26907218 10.1016/S2213-2600(16)00048-5 PMC4838193

[14] Yang WY , Rao PS, Luo YC, et al Omics-based investigation of diet-induced obesity synergized with HBx, Src, and p53 mutation accelerating hepatocarcinogenesis in zebrafish model. Cancers (Basel) 2019; 11:1899. 10.3390/cancers11121899 PMC6966430 31795276

[15] Lundberg SM , Erion G, Chen H, et al From local explanations to global understanding with explainable AI for trees. Nat Mach Intell 2020; 2:56–67. 32607472 10.1038/s42256-019-0138-9 PMC7326367

[16] US Department of Health and Human Services. National Institute of Allergy and Infectious Diseases, Division of AIDS. Division of AIDS (DAIDS) table for grading the severity of adult and pediatric adverse events, corrected version 2.1. 2018. Available at https://rsc.niaid.nih.gov/sites/default/files/daidsgradingcorrectedv21.pdf. Accessed 15 December 2021.

[17] Naranjo CA , Busto U, Sellers EM, et al A method for estimating the probability of adverse drug reactions. Clin Pharmacol Ther 1981; 30:239–45. 7249508 10.1038/clpt.1981.154

[18] Edgar R , Domrachev M, Lash AE. Gene expression omnibus: NCBI gene expression and hybridization array data repository. Nucleic Acids Res 2002; 30:207–10. 11752295 10.1093/nar/30.1.207 PMC99122

[19] Newman AM , Liu CL, Green MR, et al Robust enumeration of cell subsets from tissue expression profiles. Nat Methods 2015; 12:453–7. 25822800 10.1038/nmeth.3337 PMC4739640

[20] Lee JY , Lin SY, Lin CY, et al Identification of the PCA29 gene signature as a predictor in prostate cancer. J Bioinform Comput Biol 2019; 17:1940006. 31288639 10.1142/S0219720019400067

[21] van der Walt S , Schönberger JL, Nunez-Iglesias J, et al scikit-image: image processing in Python. PeerJ 2014; 2:e453. 25024921 10.7717/peerj.453 PMC4081273

[22] Kubat M , Matwin S. Addressing the curse of imbalanced training sets: one-sided selection. In: Proceeding of the 14th International Conference on Machine Learning. San Francisco, CA: Morgan Kaufmann Publishers, 1997: 179–186.

[23] Miller WG , Jones GRD. Estimated glomerular filtration rate; laboratory implementation and current global status. Adv Chronic Kidney Dis 2018; 25:7–13. 29499890 10.1053/j.ackd.2017.09.013

[24] Franceschini F , Bottau P, Caimmi S, et al Mechanisms of hypersensitivity reactions induced by drugs. Acta Biomed 2019; 90:44–51. 10.23750/abm.v90i3-S.8160 PMC6502177 30830061

[25] Adam J , Pichler WJ, Yerly D. Delayed drug hypersensitivity: models of T-cell stimulation. Br J Clin Pharmacol 2011; 71:701–7. 21480949 10.1111/j.1365-2125.2010.03764.x PMC3093075

[26] Loertscher R , Lavery P. The role of glycosyl phosphatidyl inositol (GPI)-anchored cell surface proteins in T-cell activation. Transpl Immunol 2002; 9:93–6. 12180852 10.1016/s0966-3274(02)00013-8

[27] Yuk JM , Silwal P, Jo EK. Inflammasome and mitophagy connection in health and disease. Int J Mol Sci 2020; 21:4714. 10.3390/ijms21134714 PMC7370205 32630319

[28] Kumar S , Jain A, Choi SW, et al Mammalian Atg8-family proteins are upstream regulators of the lysosomal system by controlling MTOR and TFEB. Autophagy 2020; 16:2305–6. 33070669 10.1080/15548627.2020.1837423 PMC7751677

[29] Moreno ML , Mérida S, Bosch-Morell F, Miranda M, Villar VM. Autophagy dysfunction and oxidative stress, two related mechanisms implicated in retinitis pigmentosa. Front Physiol 2018; 9:1008. 30093867 10.3389/fphys.2018.01008 PMC6070619

[30] Illescas O , Pacheco-Fernández T, Laclette JP, Rodriguez T, Rodriguez-Sosa M. Immune modulation by the macrophage migration inhibitory factor (MIF) family: D-dopachrome tautomerase (DDT) is not (always) a backup system. Cytokine 2020; 133:155121. 32417648 10.1016/j.cyto.2020.155121

[31] Ma L , Li F, Zhang JW, et al Host factor SPCS1 regulates the replication of Japanese encephalitis virus through interactions with transmembrane domains of NS2B. J Virol 2018; 92:e00197–00118. 29593046 10.1128/JVI.00197-18 PMC5974503

[32] Zhang R , Miner JJ, Gorman MJ, et al A CRISPR screen defines a signal peptide processing pathway required by flaviviruses. Nature 2016; 535:164–8. 27383988 10.1038/nature18625 PMC4945490

[33] Osanai T , Tanaka M, Kamada T, et al Mitochondrial coupling factor 6 as a potent endogenous vasoconstrictor. J Clin Invest 2001; 108:1023–30. 11581303 10.1172/JCI11076 PMC200946

[34] Preziosi P. Isoniazid: metabolic aspects and toxicological correlates. Curr Drug Metab 2007; 8:839–51. 18220565 10.2174/138920007782798216

