# Supplementary material for: Whole-Blood 3-Gene Signature as a Decision Aid for Rifapentine-based Tuberculosis Preventive Therapy
Source: Clin Infect Dis. 2022 Jan 5;75(5):743–52. doi: 10.1093/cid/ciac003 (PMC9477448; doi:10.1093/cid/ciac003)

**Whole-blood 3-gene signature as a Decision Aid for Rifapentine-based TB Preventive Therapy**

**Authors:** Hung-Ling Huang,* Jung-Yu Lee,* Yu-Shu Lo, I-Hsin Liu, Sing-Han Huang, Yu-Wei Huang, Meng-Rui Lee, Chih-Hsin Lee, Meng-Hsuan Cheng, Po-Liang Lu, Jann-Yuan Wang,^+^ Jinn- Moon Yang,^+^ Inn-Wen Chong

^*^ The two authors contributed equally

^+^ Corresponding authors

**Methods**

**Dosage of 3HP regimen**

Each eligible case who received a diagnosis of latent tuberculosis infection received weight-adjusted weekly 3HP regimen, which consisted of rifapentine (900 mg for participants with body weight >50.0 kg; 750 mg for 32.1–50.0 kg; 600 mg for 25.1–32.0 kg; and 450 mg for 14.1–25.0 kg) plus isoniazid (15 mg/kg, rounded up to nearest 150 mg, with a maximum of 900 mg) for a total of 12 doses. Treatment was supervised by government-paid directly observed therapy supporters.

**Laboratory procedures**

***Blood sample processing and peripheral blood mononuclear cell preparation***

Whole blood (10 mL) collected through venipuncture was centrifuged at 800 *g* for 20 minutes. Subsequently, the supernatant was isolated and frozen at −80°C. The remaining whole blood was diluted with an equal volume of sterile phosphate-buffered saline (PBS) and carefully poured onto 10 mL of Ficoll-Paque PLUS (GE Healthcare Bio-Sciences, Uppsala, Sweden). The tube was continuously centrifuged for 20 minutes at 2000 rpm (without brake). Peripheral blood mononuclear cells (PBMCs) were then harvested, washed with 5 mL of PBS, and centrifuged again for 20 minutes at 2000 rpm. PBMC pellets were suspended in ammonium-chloride-potassium lysing buffer (Invitrogen) and incubated for 10 minutes at room temperature with gentle mixing to lyse contaminated red blood cells. They were then washed with PBS ethylenediaminetetraacetic acid. Cell number and viability were determined using a Countess Automated Cell Counter (Invitrogen). In total, 1 × 10^6^ cells/mL were then resuspended in 100 µL of PBS and 1 mL of Cellbanker (Zenoaq Resource, Fukushima, Japan) and cryopreserved at −80°C.

***RNA purification***

Total RNA of each sample was extracted from 10^7^ PBMCs by using RNeasy Mini Kit (Qiagen), and then, the isolated RNAs were quantified and qualified using NanoDrop spectrophotometer (Thermo Fisher Scientific Inc.) and Agilent Bioanalyzer (Agilent Technologies, Santa Clara, CA, USA). RNA integrity (RIN) was verified through 1% formaldehyde-agarose gel electrophoresis, and the depletion of ribosomal RNA (rRNA) before RNA sequencing was applied for samples with a low RIN value initially (<5.0).

***rRNA depletion***

RNA was quantified using the NEBNext rRNA Depletion Kit (NEB #E6310). Briefly, 1 μg of the total RNA was mixed with 11 μL of formamide, 1 μL NEBNext rRNA Depletion Solution, and 2 μL probe hybridization buffer to a total volume of 15 μL. Hybridization was performed in a thermocycler with the following program: 2 minutes at 95°C, ramped down to 22°C at intervals of 0.1°C per second. After mixing with 2 μL of NEBNext RNase H, 2 μL of RNase H reaction buffer, and 1 μL of nuclease-free water to a total volume of 5 μL, the sample was incubated at 37°C for 30 minutes in a thermocycler (with lid at 40°C or off). Then, DNase I digestion was performed using the mixture of 5 μL DNase I reaction buffer, 2.5 μL DNase I (RNase-free), and 22.5 μL nuclease-free water. Thereafter, 110 μL (2.2X) resuspended RNA sample purification beads was added to the RNA sample to immobilize nucleic acid by using appropriate magnetic stand. For the first round of depletion, the hybridization reaction was added to the beads, which was incubated on ice for 15 minutes through gentle rotation followed by bead separation on a magnetic stand for 5 minutes. For the second and third rounds of depletion, the supernatant (without disturbing the beads containing the RNA) was discarded after addition of 200 μL of 80% freshly prepared ethanol. After air drying for up to 5 minutes, the beads were removed from the magnetic stand. RNA was then eluted from the beads through the addition of 8 μL nuclease-free water and collected for further next-generation sequencing library construction.

***Messenger RNA library construction and sequencing***

RNA-seq libraries were prepared with 1 μg of total RNA using Illumina TruSeq RNA-Seq v2 Kit (Illumina, San Diego, CA, USA) according to the manufacturer’s protocol. Poly(A) messenger RNA (mRNA) isolation was performed using Poly(A) mRNA Magnetic Isolation Module. The mRNA fragmentation and priming were performed using First Strand Synthesis Reaction Buffer and Random Primers. The first-strand cDNA was synthesized using ProtoScript II Reverse Transcriptase, and the second-strand cDNA was synthesized using Second Strand Synthesis Enzyme Mix. The double-stranded cDNA purified by beads was then treated with End Prep Enzyme Mix to repair both ends and add a dA-tailing in one reaction, followed by a T-A ligation to add adaptors to both ends. Size selection of adaptor-ligated DNA was then performed using beads, and fragments of approximately 420 bp (with the approximate insert size of 300 bp) were recovered. Each sample was then amplified through polymerase chain reaction (PCR) for 13 cycles by using P5 and P7 primers, with both primers carrying sequences that can anneal with the flow cell to perform bridge PCR and P7 primer carrying a six-base index allowing for multiplexing. The PCR products were cleaned up using beads, validated using a Qsep100 (BIOptic, Taiwan), and quantified using the Qubit3.0 Fluorometer (Invitrogen, Carlsbad, CA, USA).

Libraries with different indices were multiplexed, and sequencing of 150 base pair, paired-end reads was performed with an HiSeq X Ten instrument. Image analysis and base calling were conducted using the HiSeq Control Software + OLB + GAPipeline-1.6 (Illumina) on the HiSeq instrument.

***Reverse transcription-quantitative polymerase chain reaction***

Reverse transcription-quantitative polymerase chain reaction (RT-qPCR) was carried out on Applied Biosystems StepOnePlus Real-Time PCR System using Smart Quant Green Master Mix with ROX according to the manufacturer’s instructions. The thermal cycling conditions were 95°C for 10 minutes followed by 40 cycles of 95°C for 15 seconds and 60°C for 1 minute. The PCR signal specificity was confirmed using the melting curve analysis. Primers for RT-qPCR are provided in **supplementary Table S1**.

**Hierarchical system biology model**

Hierarchical system biology model (HiSBiM) is a four-level analysis, including gene, pathway, subsystem, and systems, according to Kyoto Encyclopedia of Genes and Genomes (KEGG) database [1]. The main steps of HiSBiM are differentially expressed gene (DEG) selection, pathway enrichment, and subsystem and system involvement.

***DEG selection***

The RNA sequences and quality of the 36 samples were processed and analyzed using GENEWIZ. The reads were aligned with human reference genome (GRCh38) by using the HISAT2 package (v.2.1.0) [2], the mapped reads were assembled into transcripts, and the gene abundances were estimated using the StringTie package (v.1.3.4) [3]. Next, the expression levels of all transcripts were estimated using StringTie package and calculated based on fragments per kilobase of transcript per million mapped (FPKM) by using the Ballgown package (v.2.22.0) in R (v. 3.5.3) [4]. The average mapping rate of the reads was 90%. The RNA-seq data has been deposited in National Center for Biotechnology Information Gene Expression Omnibus under accession code GSE174552 [5].

To find biomarkers for systemic drug reaction (SDR), DEGs were selected between samples before (*N_B_*) and after (*N_A_*) 3HP treatment from non-SDR group and samples before (*S_B_*) and after (*S_A_*) 3HP treatment from SDR group. First, SDR biomarker candidates were selected based on fold change (FC) ≥ 1.5 and *p* < 0.05, calculated using the Student’s *t* test for *S_B_* versus *N_B_* and *S_A_* versus *N_A_* sets.

***Pathway enrichment for DEGs***

To study how 1,243 DEGs affect SDR, the hypergeometric distribution was used to analyze pathway enrichment. We collected 322 human pathways from the KEGG database. The *p* value of the hypergeometric distribution was calculated as follows:

$$p= \sum_{i=x}^{n} \frac{(\begin{matrix} M \\ i \end{matrix})(\begin{matrix} N-M \\ n-i \end{matrix})}{(\begin{matrix} N \\ n \end{matrix})}$$

where *i* is the number of DEGs in the specific KEGG pathway, *n* is the number of genes in the specific KEGG pathway, *M* is the number of DEGs, and *N* is the number of genes recorded in the KEGG database. The *p* values were transferred to *z*-scores by using standard normal distribution.

***Subsystem and system involvement***

To understand how DEGs are involved in subsystems and systems, we proposed a HiSBiM score (*S_HiSBiM_*) for genes by calculating the meta-*z*-scores of the systems and subsystems and *z*-scores of pathways. The *S_HiSBiM_* was calculated as follows:

$$HiSBiM=Z_{Sys}+Z_{Sub}+Z_{Path}$$

where *Z_Sys_*, *Z_Sub_*, and Z*_Path_* are the meta-*z*-scores of the systems and subsystems and pathways involved in a gene, respectively. The meta-*z*-score was defined as follows:

$$Z_{Sys/Sub/Path}=\frac{\sum_{i=1}^{n} Z_{i}}{\sqrt{n}}$$

where *Z_Sys_*, *Z_Sub_*, and *Z_Path_* are the *z*-scores of the systems, subsystems, and pathways in the KEGG database; *n* is the number of systems (6), subsystems (45), or pathways (322), respectively.

**Integrated gene score for calculating the importance of DEGs**

To identify potential biomarkers, we proposed an integrated gene score (*S_IG_*) to calculate the importance of DEGs for selecting representative genes in each group. *S_IG_* consists of pathway enrichment (*S_HiSBiM_*), FC (*S_FC_*), and *p* value (*S_p_*) of a DEG. *S_IG_* is defined as follows:

$$S_{IG}=S_{HiSBiM}+S_{FC}+S_{p}$$

where *S_HiSBiM_*, *S_FC_*, and *S_p_* are computed as follows:

$$S_{HiSBiM}=\frac{HiSBiM-min({HiSBiM}_{G})}{\max\left( {HiSBiM}_{G} \right)-min({HiSBiM}_{G})} ,$$

$$S_{FC}=\frac{{log}_{2}FC-min({log}_{2}{FC}_{G})}{\max\left( {log}_{2}{FC}_{G} \right)-min({log}_{2}{FC}_{G})} ,\mathrm{and}$$

$$S_{P}=\frac{{-log}_{10}p-min(-{log}_{10}p_{G})}{\max\left( -{log}_{10}p_{G} \right)-min(-{log}_{10}p_{G})}$$

where *G* is a gene in the DEG set (here, includes 1,243 genes); max(*log_2_FC_G_*) and min(*log_2_FC_G_*) are the maximum and minimum FC of the gene G, respectively; and max(－log_10_p_G_) and min(－log_10_p_G_) are the maximum and minimum *p* values of the gene G, respectively.

**Biological similarity score for identifying representative genes**

DEGs were clustered according to our previous works,[6] and biological similarity score (*BS*) was defined as follows:

$${BS}_{i,j}=\frac{{RSS}_{{BP}_{i,j}} +{RSS}_{{CC}_{i,j}}}{2}+{PCC}_{i,j}$$

where ${RSS}_{{BP}_{i,j}}$ and ${RSS}_{{CC}_{i,j}}$ are the relative specificity similarity scores of gene ontology (GO) biological process and GO cellular component, respectively,[7] between genes *i* and *j*; *PCC_i,j_* is the Pearson’s correlation coefficient of co-expression between genes *i* and *j*.

Furthermore, DEGs were clustered based on *BS* through hierarchical clustering analysis of 60 groups with 3–127 genes in a group, and then, potential biomarkers were selected based on *S_IG_* from each group. If the number of genes in a group was <20, between 30 and 40, between 40 and 50, between 60 and 70, between 70 and 80, between 90 and 100, and >100 genes, 1, 3, 4, 6, 7, 9, and 10 genes were selected, respectively. Then, 57 potential genes were selected as gene signatures. Finally, we identified 19 potential biomarkers by integrating *BS*, *S_IG_*, and our domain knowledge, as well as further application of RT-qPCR for validation.

**Therapy–Biomarker Pathway Approach**

To determine the final representative genes, we developed a therapy–biomarker pathway approach by clustering the gene–pathway interaction similarity (*GP*). On the basis of the selected 1,243 DEGs, we used the hypergeometric distribution to compute the enrichment of KEGG pathways. Next, we calculated the gene–pathway similarity of the gene pair *x* and *y* by using weighted Jaccard coefficient (*GP_j_*) according to respective vectors, x = (*x_1_*, *x_2_*,…, *x_n_*) and y = (*y_1_*, *y_2_*,…, *y_n_*), of pathway enrichments. The *GP_j_* is computed as follows:

$${GP}_{j}(\text{x},\text{y})=\frac{\sum_{i=1}^{n} min(x_{i},y_{i})}{\sum_{i=1}^{n} max(x_{i},y_{i})}$$

where *n* is the total number of enriched pathways (*p*<0.05). Then, we clustered the *p* value of 33 enriched KEGG pathways across 19 potential biomarkers based on hierarchical clustering using the complete linkage method with Euclidean distance (supplementary figure S1). By integrating *BS*, *S_IG_*, and *GP* scores, we determined six represented genes for the SDR predictive model construction by using random forest models and SHapley Additive ExPlanation (SHAP) values.

**Shapley Additive Explanation**

Here, we used an enhanced interpretability of tree-based models, called TreeExplainer [8] based on the classic game-theoretic SHAP value [9-11], which can interpret the contribution of features to a prediction model. TreeExplainer offered three advantages: (1) providing the exact SHAP values in polynomial time; (2) supplying the direct measures of local feature interactions; and (3) providing the interpretation of the global model structure through the combination of numerous local explanations.

**Results**

**Cases enrolled in previous studies**

Among the 187 cases enrolled, the clinical data of 46 subjects [12] and plasma concentrations of isoniazid and rifapentine and genotypes of drug metabolizing enzymes of the two drugs of another 13 cases [13] have been reported in previous studies.

**Therapy-biomarker pathway of the 19 potential biomarker gens identified in the pilot cohort**

When *M. tuberculosis*, along with rifapentine and isoniazid enters the macrophages, they might modulate the function of phagosomes (e.g., *ATP6V0E1*, *RAB11B*, *ARPC2*, and *TUBA1C*) [14]. Immune responses are then activated through immune-related genes, such as *GABARAPL2* and *PYCARD*, leading to cytokine storm and organ damage (e.g., *DDT*). Simultaneously, the oxidative capacity of mitochondria increases to accelerate the breaking down of pathogens (e.g., *ATP5PF* and *NDUFB11*) [15]. All of the aforementioned alteration may affect the central dogma process, including *PIGX*, *ATF4*, *SSU72*, *HNRNPC*, *HDAC1*, *POLR2J*, *MANF*, *RPL27A*, and *SUMO1*, and protein transport (e.g., *SPCS1*).

**Table S1: Sequences of sense and antisense primers and positive controls in reverse transcription-quantitative polymerase chain reaction**

| **Gene name** | **Transcript name** | **Positive control** | **Forward primer** | **Reverse primer** |
| --- | --- | --- | --- | --- |
| ATP5PF | NM_001003703 | HeLa | TCGGGACTGAGTGCAAGAAT | AACACCAATGTTCCTCCGCA |
| SPCS1 | NM_014041 | HeLa | TGGATTACAAGGGCCAGAAG | GCCACGTACCCGTAGATAAAT |
| ATF4 | NM_182810 | HEK293 | GTCCCTCCAACAACAGCAAG | CTATACCCAACAGGGCATCC |
| GABARAPL2 | NM_007285 | HEK293 | AGCTTCCTTCTGAAAAGGCG | TCTCTCCGCTGTAGGCCACA |
| HDAC1 | NM_004964 | HEK293 | TATTATGGACAAGGCCACCC | CATCTCCTCAGCATTGGCTT |
| MANF | NM_006010 | HEK293 | GTGCACGGACCGATTTGTAG | GGAAAGCTCCAGGCTTCACA |
| NDUFB11 | NM_019056 | HEK293 | ACGTCTGGAACATGCGACTT | CTTTCATCCCATCCCACGCT |
| RAB11B | NM_004218 | HEK293 | TTCACCCGCAACGAGTTCAA | AGATCTGCGCCTTGATGGTC |
| RPL27A | NM_000990 | HEK293 | GGGAAAGGGAAAGCTCCCAA | CTCCCTCCATGTGGCTTCAA |
| SSU72 | NM_014188 | HEK293 | TCCTCAGCAAACGGGGATTC | AAACATTGGGCTTGTCGGGA |
| SUMO1 | NM_003352 | HEK293 | AGCAGTGAGATTCACTTCAAAGTG | TCTGACCCTCAAAGAGAAACCTG |
| DDT | NM_001084392 | HEK293 | GAGTTTCTCACCAAGGAGCTAGC | AAGCAGCCAGTTCACAGATGCC |
| ARPC2 | NM_152862 | MCF7 | GAACCTCCTCTGGAGCTGAAAG | GAACGTGTGGATCAGGTTGATGG |
| ATP6V0E1 | NM_003945 | MCF7 | TGACCTGTTCAGTTTGCTGC | CATGTCTTCTTCCTCAAGGC |
| PIGX | NM_017861 | MCF7 | CTGTGCACTGCCGCTATCAT | ATCGGGAACTCTTGGTCACA |
| POLR2J | NM_006234 | MCF7 | CAGGAAGCCTTTACCAACGCCA | CACAGGTAGGAACGGGGCTCA |
| TUBA1C | NM_032704 | MCF7 | TCAACACCTTCTTCAGTGAAACG | AGTGCCAGTGCGAACTTCATC |
| HNRNPC | NM_031314 | MCF7 | GATGTACGGGTCAGTAACAGAAC | AGCCCGAGCAATAGGAGGA |
| PYCARD | NM_013258 | MCF7 | CCATCCTAGAGGCACTGGAA | CTCCGTACGCCTCCAGATAG |
| GAPDH | NM_002046 |  | GTCTCCTCTGACTTCAACAGCG | ACCACCCTGTTGCTGTAGCCAA |

**Table S2. Characteristics of participants with systemic drug reaction (SDR)**

|  | **Pilot cohort**  **(n=8)** | **Training cohort**  **(n=28)** | **Testing Cohort**  **(n=8)** |
| --- | --- | --- | --- |
| Age (year) | 51.2 ± 10.8 | 47.7 ± 14.1 | 48.1 ± 14.0 |
| ≤35 | 1 (13%) | 6 (21%) | 2 (25%) |
| >35 | 7 (88%) | 22 (79%) | 6 (75%) |
| Female sex | 6 (75%) | 17 (61%) | 4 (50%) |
| Body-mass index (kg/m^2^) | 22.6 ± 2.7 | 23.7 ± 3.3 | 24.0 ± 3.6 |
| eGFR (ml/min/1.73^2^) | 105.3 ± 29.5 | 102.3 ± 36.9 | 99.3 ± 18.4 |
| INH/RPT dose (mg/kg/dose) | 15.1 ± 1.8 | 14.4 ± 2.5 | 14.2 ± 2.2 |
| SDR type |  |  |  |
| Flu-like syndrome | 8 (100%) | 23 (82%)**^†^** | 7 (88%)**^‡^** |
| Hypotension | 2 (25%)* | 2 (7%) | 1 (13%) |
| Urticaria | 1 (13%)* | 4 (14%) | 0 |
| Conjunctivitis | 0 | 2 (7%) | 1 (13%) |
| Bronchospasm | 0 | 0 | 1 (13%) |
| Angioedema | 0 | 1 (4%) | 0 |
| Onset dose | 3 [3.0 – 4.3] | 3 [3.0 – 4.0] | 3 [2.5 – 3.0] |
| Onset after medications (hour) | 5 [2.6 – 5.3] | 4 [2.8 – 6.0] | 5 [2.6 – 5.5] |
| Duration of symptoms (hour) | 29 [22.0 – 33.0] | 24 [18.0 – 30.0] | 18 [12.0 – 30.0] |
| Severity of SDR |  |  |  |
| Grade 3 | 1 (13%) | 3 (11%) | 2 (25%) |
| Grade 2 | 7 (88%) | 25 (89%) | 6 (75%) |
| Permanent discontinuation | 4 (50%) | 14 (50%) | 3 (38%) |

Data are presented as number (percentage), mean ± standard deviation, or median [interquartile range].

eGFR: estimated glomerular filtration rate; INH: isoniazid; RPT: rifapentine; SDR: systemic drug reaction.

*Two cases with hypotension and one case had urticaria had flu-like syndrome concomitantly.

**^†^** Two cases had hypotension, 1 case had urticaria and 1 case had conjunctivitis, concomitantly.

**^‡^**One case had hypotension and one case had bronchospasm concomitantly.

**Table S3. Detailed information on adverse events in cases with and without SDR**

|  | Pilot cohort | |  | Training cohort | |  | Testing cohort | |
| --- | --- | --- | --- | --- | --- | --- | --- | --- |
|  | **SDR**  **(n=8)** | **non-SDR**  **(n=12)** |  | **SDR**  **(n=28)** | **non-SDR**  **(n=104)** |  | **SDR**  **(n=8)** | **non-SDR**  **(n=27)** |
| Grade 3 SDR | 1 (12.5%) | 0 |  | 3 (10.7%) | 0 |  | 2 (25.0%) | 0 |
| Any flu-like symptoms | 1 (12.5%) | 0 |  | 3 (10.7%) | 1 (1.0%) |  | 2 (25.0%) | 0 |
| Hypotension | 1 (12.5%) | 0 |  | 2 (7.1%) | 0 |  | 1 (12.5%) | 0 |
| Fever | 1 (12.5%) | 0 |  | 3 (10.7%) | 0 |  | 2 (25.0%) | 0 |
| Angioedema | 0 | 0 |  | 1 (3.6%) | 0 |  | 0 | 0 |
| Bronchospasm | 0 | 0 |  | 0 | 0 |  | 1 (12.5%) | 0 |
| Shortness of breath | 0 | 0 |  | 0 | 1 (1.0%) |  | 0 | 0 |
| Grade 2 SDR | 7 (87.5%) | 0 |  | 25 (89.3%) | 0 |  | 6 (75.0%) | 0 |
| Any flu-like symptoms | 7 (87.5%) | 1 (8.3%) |  | 20 (71.4%) | 17 (16.2%) |  | 5 (62.5%) | 3 (11.1%) |
| Fever | 7 (87.5%) | 1 (8.3%) |  | 18 (64.3%) | 14 (13.5%) |  | 5 (62.5%) | 3 (11.1%) |
| Headache | 3 (37.5%) | 0 |  | 3 (10.7%) | 0 |  | 2 (25.0%) | 0 |
| Chills and flush | 2 (25.0%) | 1 (8.3%) |  | 3 (10.7%) | 6 (5.8%) |  | 1 (12.5%) | 2 (7.4%) |
| Myalgia | 2 (25.0%) | 0 |  | 7 (25.0%) | 0 |  | 2 (25.0%) | 0 |
| Malaise | 1 (12.5%) | 0 |  | 0 | 1 (1.0%) |  | 0 | 0 |
| Dizziness | 2 (25.0%) | 0 |  | 2 (7.1%) | 2 (1.9%) |  | 2 (25.0%) | 1 (3.7%) |
| Nausea/vomiting | 1 (12.5%) | 0 |  | 1 (3.6%) | 2 (1.9%) |  | 1 (12.5%) | 0 |
| Cough | 1 (12.5%) | 0 |  | 1 (3.6%) | 0 |  | 0 | 0 |
| Epigastralgia | 1 (12.5%) | 0 |  | 2 (7.1%) | 4 (3.8%) |  | 1 (12.5%) | 1 (3.7%) |
| Urticaria | 1 (12.5%) | 0 |  | 4 (14.3%) | 0 |  | 0 | 0 |
| Conjunctivitis | 0 |  |  | 2 (7.1%) |  |  | 1 (12.5%) | 0 |
| Skin rash/itching | 0 | 1 (8.3%) |  | 1 (3.6%) | 3 (2.9%) |  | 1 (12.5%) | 0 |
| Diarrhea | 0 | 0 |  | 0 | 1 (1.0%) |  | 0 | 0 |
| Hepatotoxicity | 1 (12.5%) | 0 |  | 2 (7.1%) | 0 |  | 0 | 1 (3.7%) |
| Grade 1 |  |  |  |  |  |  |  |  |
| Hypotension | 0 | 0 |  | 1 (3.6%) | 0 |  | 0 | 0 |
| Fever | 0 | 0 |  | 2 (7.1%) | 3 (2.9%) |  | 1 (12.5%) | 1 (3.7%) |
| Limb edema | 0 | 0 |  | 1 (3.6%) | 0 |  | 0 | 1 (3.7%) |
| Dizziness | 3 (37.5%) | 3 (25.0%) |  | 9 (32.1%) | 15 (14.4%) |  | 5 (62.5%) | 2 (7.4%) |
| Malaise | 4 (50.0%) | 2 (16.7%) |  | 11 (39.3%) | 13 (12.5%) |  | 4 (50.0%) | 5 (18.5%) |
| Myalgia | 5 (62.5%) | 3 (25.0%) |  | 9 (32.1%) | 13 (12.5%) |  | 3 (37.5%) | 4 (14.8%) |
| Abdominal discomfort | 2 (25.0%) | 2 (16.7%) |  | 0 | 8 (7.7%) |  | 2 (25.0%) | 0 |
| Anorexia | 1 (12.5%) | 1 (8.3%) |  | 6 (21.4%) | 3 (2.9%) |  | 2 (25.0%) | 1 (3.7%) |
| Nausea | 1 (12.5%) | 1 (8.3%) |  | 7 (25.0%) | 14 (13.5%) |  | 4 (50.0%) | 1 (3.7%) |
| Headache | 4 (50.0%) | 1 (8.3%) |  | 7 (25.0%) | 13 (12.5%) |  | 2 (25.0%) | 1 (3.7%) |
| Cough | 1 (12.5%) | 1 (8.3%) |  | 0 | 0 |  | 0 | 0 |
| Febrile sensation/Chills | 4 (50.0%) | 1 (8.3%) |  | 8 (28.6%) | 12 (11.5%) |  | 3 (37.5%) | 3 (11.1%) |
| Irregular menstruation | 0 | 1 (8.3%) |  | 0 | 1 (1.0%) |  | 0 | 0 |
| Cutaneous reactions | 2 (25.0%) | 1 (8.3%) |  | 0 | 2 (1.9%) |  | 1 (12.5%) | 2 (7.4%) |
| Palpitation | 2 (25.0%) | 1 (8.3%) |  | 3 (10.7%) | 0 |  | 1 (12.5%) | 0 |
| Diarrhea | 1 (12.5%) | 1 (8.3%) |  | 0 | 3 (2.9%) |  | 0 | 0 |
| Limb numbness | 0 | 1 (8.3%) |  | 0 | 1 (1.0%) |  | 0 | 0 |
| Hepatotoxicity | 0 | 0 |  | 0 | 3 (2.9%) |  | 0 | 1 (3.7%) |

Data are presented as number (percentage).

The denominator in the calculation of each percentage is the number of individuals in the corresponding SDR and non-SDR groups.

SDR: systemic drug reaction.

**Table S4. Characteristics of cases for priority testing of reverse transcription-quantitative polymerase chain reaction**

|  | Overall  (n=5) | SDR  (n=3) | non-SDR  (n=2) |
| --- | --- | --- | --- |
| Age (year) | 51.0 ± 17.4 | 58.5 ± 8.7 | 39.6 ± 25.2 |
| ≤35 | 1 (20%) | 0 | 1 (50%) |
| >35 | 4 (80%) | 3 (100%) | 1 (50%) |
| Female sex | 3 (60%) | 3 (100%) | 0 |
| Body-mass index (kg/m^2^) | 25.1 ± 2.2 | 25.7± 3.0 | 24.4 ± 0.6 |
| Diabetes mellitus | 1 (20%) | 0 | 1 (50%) |
| Hypertension | 1 (20%) | 0 | 1 (50%) |
| Autoimmune | 0 | 0 | 0 |
| Isoniazid/Rifapentine dose (mg/kg) | 13.6 ± 2.5 | 15.1 ± 2.2 | 11.5 ± 0.9 |
| Hemoglobin (g/dL) | 13.4 ± 2.1 | 13.3 ± 1.5 | 13.5 ± 3.5 |
| Leukocyte (K/uL) | 7.1 ± 1.1 | 7.5 ± 1.7 | 6.5 ± 3.0 |
| Platelet (K/uL) | 261 ± 113 | 298 ± 22 | 205 ± 199 |
| Alanine transaminase (U/L) | 17.0 ± 7.1 | 17.0 ± 7.2 | 17.0 ± 9.9 |
| eGFR (mL/min/1.73m^2^) | 95 ± 19 | 85 ± 11 | 112 ± 17 |
| QuantiFERON response (IU/mL)* | 2.4 ± 2.0 | 1.7 ± 0.6 | 3.5 ± 3.2 |
| Any adverse event during 3HP | 4 (80%) | 3 (100%) | 1 (50%) |
| Grade 3 | 0 | 0 | 0 |
| Grade 2 | 3 (60%) | 3 (100%)**^†^** | 0 |
| Grade 1 | 1 (20%) | 0 | 1 (50%)**^‡^** |

eGFR: estimated glomerular filtration rate; SDR: systemic drug reaction.

Data are presented as number (percentage) or mean ± standard deviation.

No statistically significant intergroup differences exist among the variables.

*QuantiFERON response was defined as the difference between interferon-gamma level of Antigen and Nil tubes by using the QuantiFERON-TB Gold in-Tube test (Cellestis/Qiagen, Carnegie, Australia).

**^†^**Grade 2 adverse drug reactions included fever, myalgia, malaise, epigastralgia, and vomiting.

**^‡^**Grade 1 adverse drug reaction was dizziness.

**Table S5. Information on the six mRNA genes for constructing SDR models**

| **Gene Name** | **Gene ID** | **Chromosome** | **Transcript Name** | **UniProt** | **Function** |
| --- | --- | --- | --- | --- | --- |
| ATP5PF | 522 | 21q21.3 | NM_001003703 | P18859 | Mitochondrial ATP synthase; catalyzes ATP synthesis |
| PIGX | 54965 | 3q29 | NM_017861 | Q8TBF5 | Encodes a type I transmembrane protein in the endoplasmic reticulum, which is related to glycosylphosphatidylinositol (GPI)-anchor biosynthesis. |
| SPCS1 | 28972 | 3p21.1 | NM_014041 | Q9Y6A9 | Component of the microsomal signal peptidase complex, which removes signal peptides from nascent proteins as they are translocated into the lumen of the endoplasmic reticulum. |
| DDT | 1652 | 22q11.23 | NM_001084392 | P30046 | Tautomerization of D-dopachrome with decarboxylation to give 5,6-dihydroxyindole. |
| GABARAPL2 | 11345 | 16q23.1 | NM_007285 | P60520 | Modulates intra-Golgi transport through coupling between N-ethylmaleimide-sensitive factor (NSF) activity and soluble NSF attachment protein receptors (SNAREs) activation. Involved in autophagy. Plays a role in mitophagy, which contributes to the regulation of mitochondrial quantity and quality through the elimination of mitochondria to a basal level to fulfill cellular energy requirements and prevents excess production of reactive oxygen species (ROS). |
| ATP6V0E1 | 8992 | 5q35.1 | NM_003945 | O15342 | Vacuolar ATPase is an enzyme transporter that functions to acidify intracellular compartments in eukaryotic cells. It is ubiquitously expressed and is present in endomembrane organelles, such as vacuoles, lysosomes, and endosomes. |

ATP: adenosine triphosphate; ATP5PF: ATP synthase peripheral stalk subunit F6; ATP6V0E1: ATPase H+ transporting V0 subunit e1; DDT: D-dopachrome tautomerase; GABARAPL2: GABA type A receptor associated protein like 2; PIGX: phosphatidylinositol glycan anchor biosynthesis class X; SDR: systemic drug reaction; SPCS1: signal peptidase complex subunit 1.

**Table S6. Performance of the 14 top-ranked models built with the expression of the six identified genes to predict systemic drug reaction in the training cohort**

| **Model** | **G-mean** | **Sensitivity** | **Specificity** |
| --- | --- | --- | --- |
| ATP6V0E1-SPCS1-GABARAPL2 | 0.985 | 1.000 | 0.971 |
| ATP5PF-ATP6V0E1-PIGX-SPCS1-DDT | 0.985 | 1.000 | 0.971 |
| **ATP6V0E1-PIGX-SPCS1** | 0.981 | 1.000 | 0.962 |
| ATP6V0E1-PIGX-SPCS1-GABARAPL2 | 0.981 | 1.000 | 0.962 |
| **ATP5PF-ATP6V0E1-PIGX-SPCS1** | 0.981 | 1.000 | 0.962 |
| ATP5PF-SPCS1-DDT | 0.981 | 1.000 | 0.962 |
| **ATP6V0E1-PIGX-SPCS1-DDT** | 0.976 | 1.000 | 0.952 |
| PIGX-SPCS1-GABARAPL2 | 0.976 | 1.000 | 0.952 |
| ATP5PF-ATP6V0E1-SPCS1-GABARAPL2-DDT | 0.976 | 1.000 | 0.952 |
| SPCS1-GABARAPL2-DDT | 0.976 | 1.000 | 0.952 |
| **ATP6V0E1-SPCS1-DDT** | 0.976 | 1.000 | 0.952 |
| ATP5PF-ATP6V0E1-PIGX-SPCS1-GABARAPL2 | 0.976 | 1.000 | 0.952 |
| ATP6V0E1-PIGX-SPCS1-GABARAPL2-DDT | 0.976 | 1.000 | 0.952 |
| ATP6V0E1-SPCS1 | 0.976 | 1.000 | 0.952 |

**Table S7: Performance of the four selected prediction models for systemic drug reaction in the testing cohort**

| **Model** | **G-mean** | **Sensitivity** | **Specificity** |
| --- | --- | --- | --- |
| **ATP6V0E1-PIGX-SPCS1** | 0.882 | 0.875 | 0.889 |
| ATP5PF-ATP6V0E1-PIGX-SPCS1 | 0.799 | 0.750 | 0.852 |
| **ATP6V0E1-PIGX-SPCS1-DDT** | 0.882 | 0.875 | 0.889 |
| ATP6V0E1-SPCS1-DDT | 0.805 | 0.875 | 0.741 |

**References**

1. Kanehisa M, Goto S. KEGG: kyoto encyclopedia of genes and genomes. Nucleic Acids Res*,* **2000** ; 28: 27-30.

2. Pertea M, Kim D, Pertea GM*, et al.* Transcript-level expression analysis of RNA-seq experiments with HISAT, StringTie and Ballgown. Nat Protoc, **2016** ; 11: 1650-1667.

3. Pertea M, Pertea GM, Antonescu CM*, et al.* StringTie enables improved reconstruction of a transcriptome from RNA-seq reads. Nat Biotechnol, **2015** ; 33: 290-295.

4. Frazee AC, Pertea G, Jaffe AE*, et al.* Ballgown bridges the gap between transcriptome assembly and expression analysis. Nat Biotechnol, **2015** ; 33: 243-246.

5. Edgar R, Domrachev M, Lash AE. Gene Expression Omnibus: NCBI gene expression and hybridization array data repository. Nucleic Acids Res, **2002** ; 30: 207-210.

6. Lee JY, Lin SY, Lin CY*, et al.* Identification of the PCA29 gene signature as a predictor in prostate cancer. J Bioinform Comput Biol, **2019** ; 17: 1940006.

7. Wu X, Zhu L, Guo J*, et al.* Prediction of yeast protein-protein interaction network: insights from the Gene Ontology and annotations. Nucleic Acids Res, **2006** ; 34: 2137-2150.

8. Lundberg SM, Erion G, Chen H*, et al.* From Local Explanations to Global Understanding with Explainable AI for Trees. Nat Mach Intell*,* **2020** ; 2: 56-67.

9. Datta A, Sen S, Zick Y. Algorithmic Transparency via Quantitative Input Influence: Theory and Experiments with Learning Systems. In: 2016 IEEE Symposium on Security and Privacy (SP); **2016**: 598-617.

10. Štrumbelj E, Kononenko I. Explaining prediction models and individual predictions with feature contributions. Knowl Inf Syst, **2014** ; 41: 647-665.

11. Shapley LS. A value for n-person games. Contrib Theor Games, **1953** ; 2: 307-317.

12. Huang HL, Lee MR, Cheng MH*, et al.* Impact of age on outcome of rifapentine-based weekly therapy for latent tuberculosis infection. Clin Infect Dis, **2021** ; 73(5): e1064-e1071.

13. Lee MR, Huang HL, Lin SW*, et al.* Isoniazid Concentration and NAT2 Genotype Predict Risk of Systemic Drug Reactions during 3HP for LTBI. J Clin Med*,* **2019** ; 8: 812.

14. Russell DG. Mycobacterium tuberculosis: here today, and here tomorrow. Nat Rev Mol Cell Biol, **2001** ; 2: 569-577.

15. West AP, Shadel GS. Mitochondrial DNA in innate immune responses and inflammatory pathology. Nat Rev Immunol, **2017** ; 17: 363-375.

**Figure S1. Heatmap and hierarchical clustering of 33 enriched pathways for 19 potential biomarkers.** The 19 genes could significantly regulate four types of pathways, including neural-related (pink), immune-related (yellow), infection-related (blue), and metabolism-related (brown). Among these genes, the six selected potential biomarkers are marked in red. Hierarchical cluster analysis was performed using Euclidean distance (complete linkage).

**
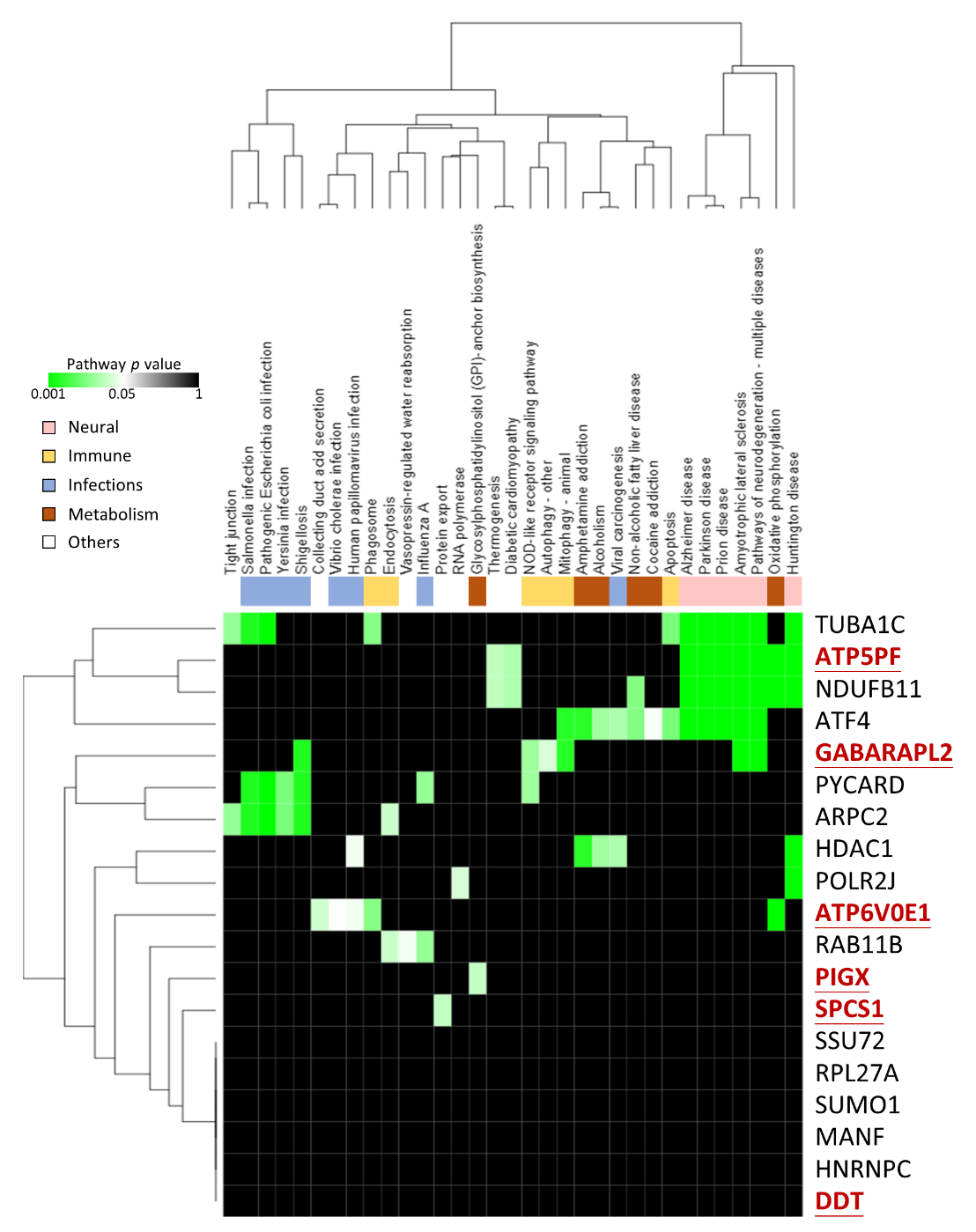
**

**Figure S2. Gene expression of 13 potential signatures in a part of training samples.** Bar chart illustrating the gene expression of *ARPC2*, *ATF4*, *HDAC1*, *HNRNPC*, *MANF*, *NDUFB11*, *POLR2J*, *PYCARD*, *RAB11B*, *RPL27A*, *SSU72*, *SUMO1*, and *TUBA1C* in three SDR (red) and two non-SDR (blue) samples collected before 3HP treatment. The expression levels were validated through reverse transcription-quantitative polymerase chain reaction.

**
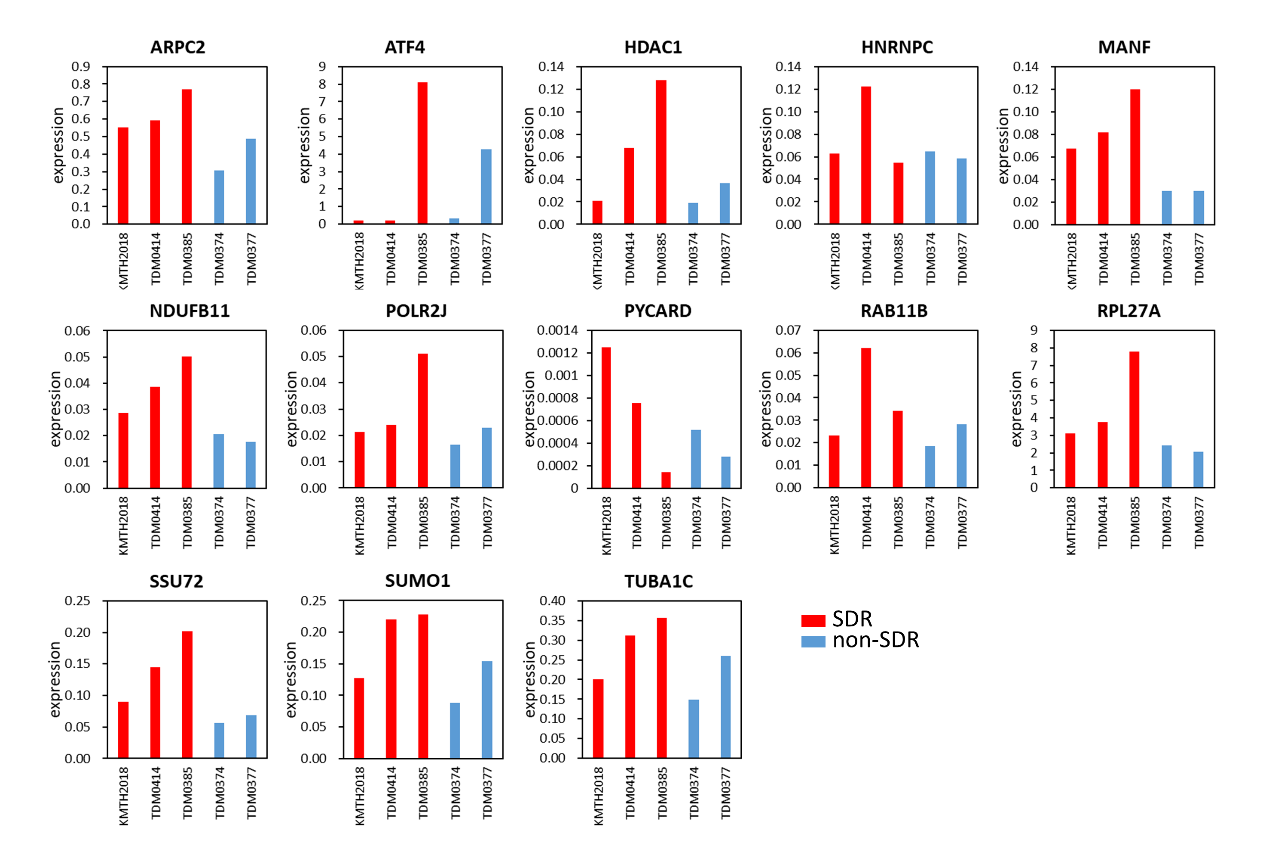
**

**Figure S3. Peripheral blood immune cell subtypes analyzed using CIBERSORT for samples from the pilot cohort.** Bar chart demonstrating the 10 immune cell subtypes of pretreatment samples from 8 SDR (red) and 8 non-SDR (blue) participants in the pilot cohort. The *p* values were calculated using Mann–Whitney *U* test.

**
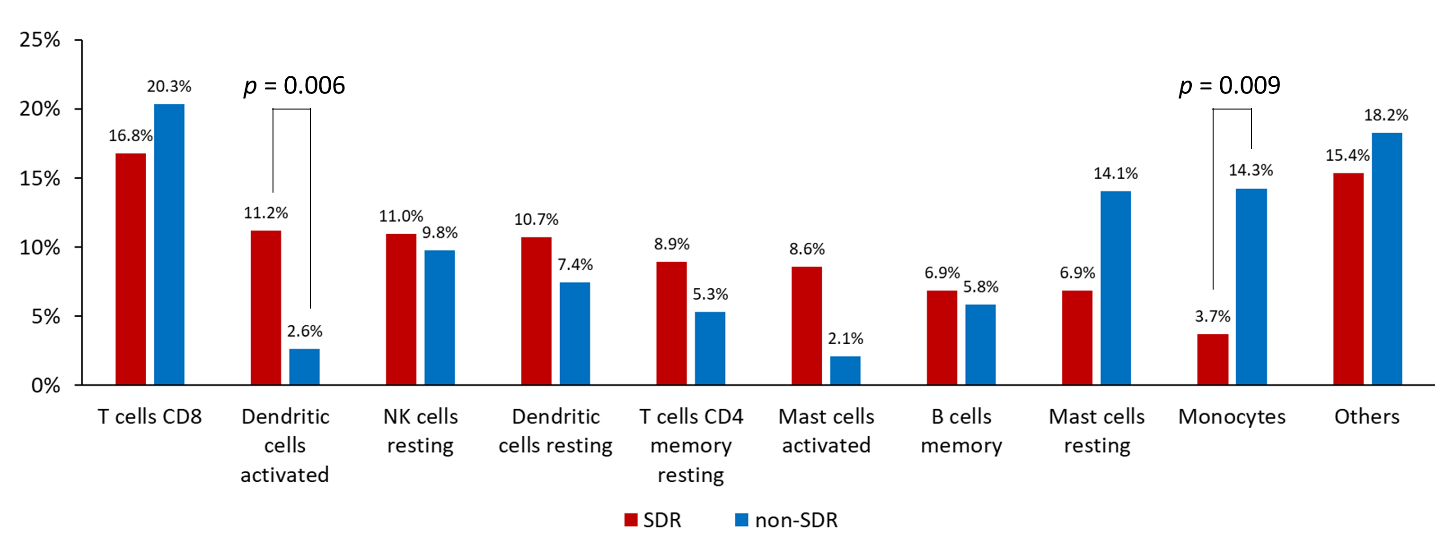
**

**Figure S4. Heat map of similarities among six identified genes in 132 samples from the training cohort, stratified by SDR occurrence.** Heat map for similarities in gene expression among 28 SDR (orange) and 104 non-SDR samples (purple). Hierarchical cluster analysis was performed using Euclidean distance (average linkage).


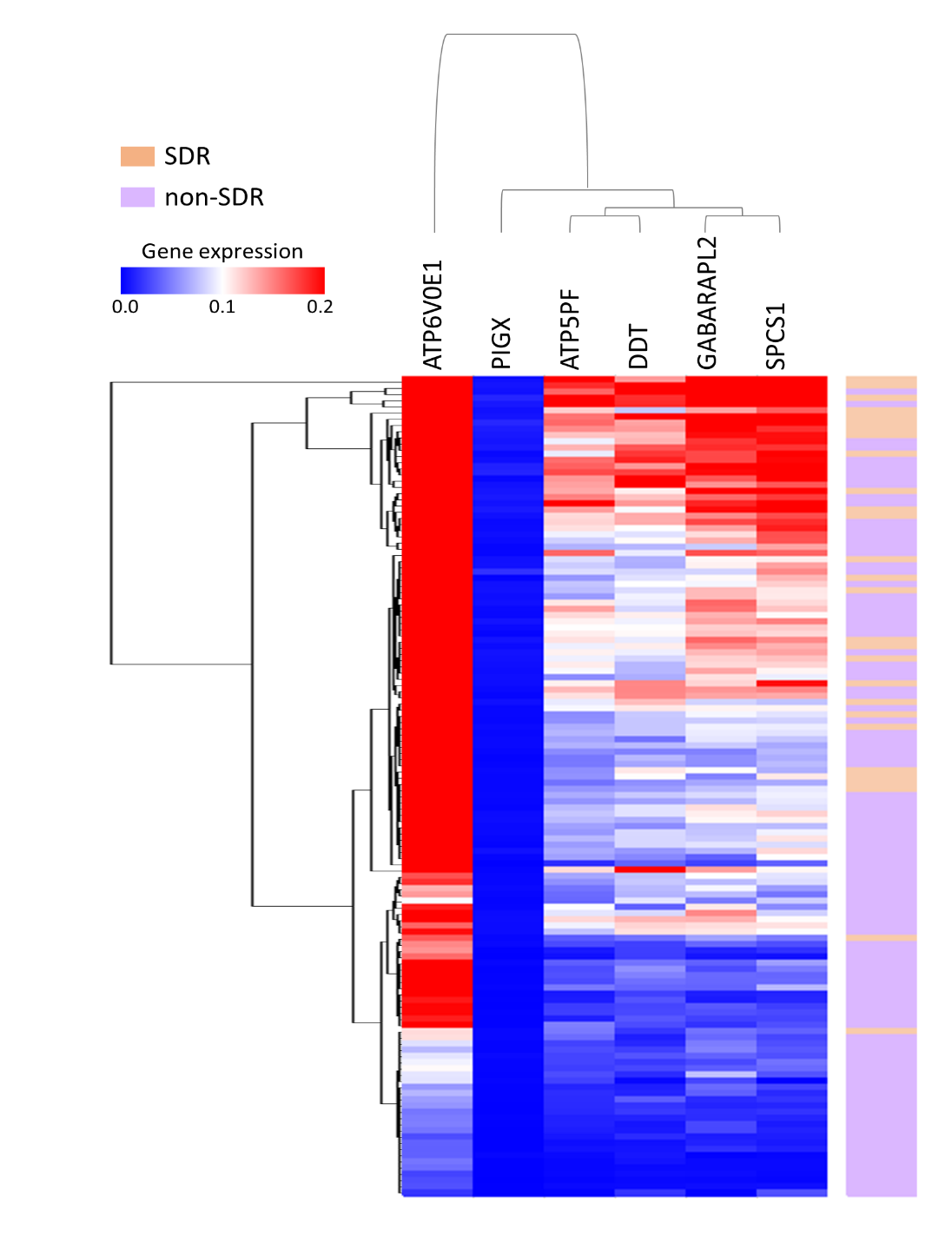


**Figure S5. SDR predictive models with universal cutoff values in the training cohort.** Interpretation of four SDR predictive models for the 28 SDR (orange) and 104 non-SDR (blue) training samples: **A)** ATP6V0E-PIGX-SPCS1, **B)** ATP5PF-ATP6V0E1-PIGX-SPCS1, **C)** ATP6V0E1-PIGX-SPCS1-DDT, and **D)** ATP6V0E1-SPCS1-DDT. SHAP output values of each sample are shown as red diamonds, and the universal cutoff value is 0 (deep red line).


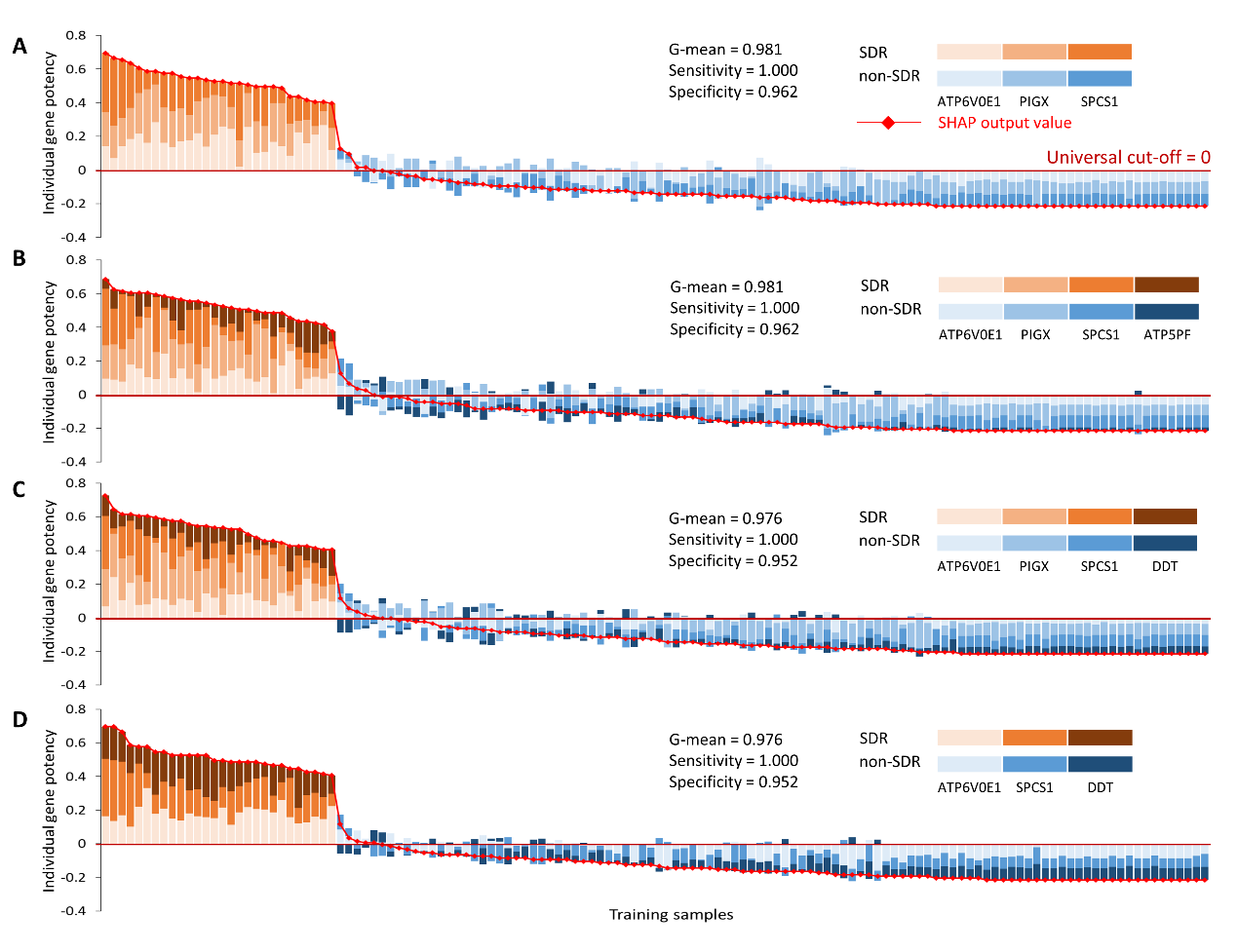


**Figure S6. Gene expression of six selected genes in the testing cohort.** Boxplot of the gene expression of *ATP5PF*, *ATP6V0E1*, *PIGX*, *SPCS1*, *GABARAPL2*, and *DDT* in 8 SDR (pink) and 27 non-SDR (blue) testing samples. Boxes indicate the sample median and interquartile range, whereas bars and colored dots indicate the range and outliers, respectively. The data were analyzed using Mann–Whitney *U* test.


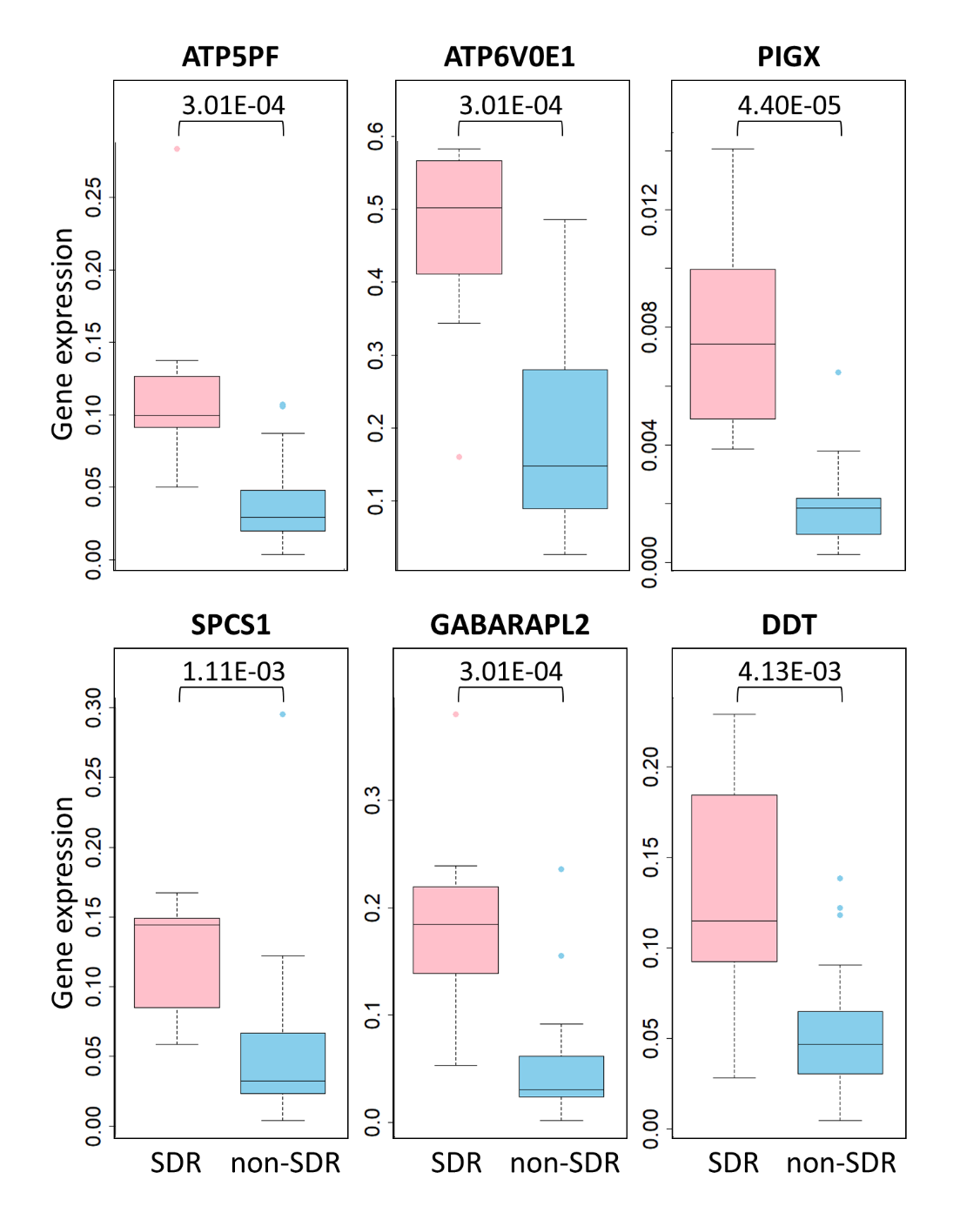

Supplement: ciac003_suppl_Supplementary_Material [file ciac003_suppl_supplementary_material.docx]
